# Prevalence and predictors of death and severe disease in patients hospitalized due to COVID-19: A comprehensive systematic review and meta-analysis of 77 studies and 38,000 patients

**DOI:** 10.1371/journal.pone.0243191

**Published:** 2020-12-07

**Authors:** Kunchok Dorjee, Hyunju Kim, Elizabeth Bonomo, Rinchen Dolma

**Affiliations:** 1 School of Medicine Division of Infectious Diseases, Center for TB Research, Johns Hopkins University, Baltimore, Maryland, United States of America; 2 Department of Epidemiology, Bloomberg School of Public Health, Johns Hopkins University, Baltimore, Maryland, United States of America; 3 Center for Alcohol and Addiction Studies, Brown University School of Public Health, Brown University, Providence, Rhode Island, United States of America; Universita degli Studi Magna Graecia di Catanzaro, ITALY

## Abstract

**Introduction:**

Progression of COVID-19 to severe disease and death is insufficiently understood.

**Objective:**

Summarize the prevalence of risk factors and adverse outcomes and determine their associations in COVID-19 patients who were hospitalized.

**Methods:**

We searched Medline, Embase and Web of Science for case-series and observational studies of hospitalized COVID-19 patients through August 31, 2020. Data were analyzed by fixed-effects meta-analysis using Shore’s adjusted confidence intervals to address heterogeneity.

**Results:**

Seventy-seven studies comprising 38906 hospitalized patients met inclusion criteria; 21468 from the US-Europe and 9740 from China. Overall prevalence of death [% (95% CI)] from COVID-19 was 20% (18–23%); 23% (19–27%) in the US and Europe and 11% (7–16%) for China. Of those that died, 85% were aged≥60 years, 66% were males, and 66%, 44%, 39%, 37%, and 27% had hypertension, smoking history, diabetes, heart disease, and chronic kidney disease (CKD), respectively. The case fatality risk [%(95% CI)] were 52% (46–60) for heart disease, 51% (43–59) for COPD, 48% (37–63) for chronic kidney disease (CKD), 39% for chronic liver disease (CLD), 28% (23–36%) for hypertension, and 24% (17–33%) for diabetes. Summary relative risk (sRR) of death were higher for age≥60 years [sRR = 3.6; 95% CI: 3.0–4.4], males [1.3; 1.2–1.4], smoking history [1.3; 1.1–1.6], COPD [1.7; 1.4–2.0], hypertension [1.8; 1.6–2.0], diabetes [1.5; 1.4–1.7], heart disease [2.1; 1.8–2.4], CKD [2.5; 2.1–3.0]. The prevalence of hypertension (55%), diabetes (33%), smoking history (23%) and heart disease (17%) among the COVID-19 hospitalized patients in the US were substantially higher than that of the general US population, suggesting increased susceptibility to infection or disease progression for the individuals with comorbidities.

**Conclusions:**

Public health screening for COVID-19 can be prioritized based on risk-groups. Appropriately addressing the modifiable risk factors such as smoking, hypertension, and diabetes could reduce morbidity and mortality due to COVID-19; public messaging can be accordingly adapted.

## Introduction

Coronavirus disease-19 (COVID-19) caused by severe acute respiratory syndrome- coronavirus-2 (SARS-CoV-2) that first emerged in Wuhan, China in late December 2019 has spread with such rapidity and efficiency that in less than 10 months, it has caused more than 36 million cases and million deaths globally [1]. Driven by an urgency to solve the crisis, studies are being published at an unprecedented pace. However, across the publications, prevalence of death, severe disease and their association with epidemiological risk factors have greatly varied [2, 3], with studies showing conflicting results for association of key risk factors such as sex [4–8], smoking [9–12], hypertension [4, 7, 8, 13, 14] and diabetes [4, 7, 8, 13, 14] with COVID-19 disease severity and death. Whether or how cardiovascular risk factors, especially prior hypertension, diabetes and heart disease are associated with acquisition of SARS-CoV-2 and progression to severe disease or death is not understood well [15–18]. Meta-analyses conducted so far on prevalence of epidemiological risk factors and association with disease progression were mostly based on studies from China [9, 11, 18–20] and many of the analyses on prevalence estimates included studies focused on critically ill patients [9, 19], which can overestimate the prevalence and affect generalizability of results. To our knowledge, none of the analyses were restricted to hospitalized COVID-19 patients. Restricting our analysis to hospitalized patients provides an efficient sampling frame to investigate disease progression in relation to risk factors.

Therefore, we undertook a comprehensive systematic review and meta-analysis to investigate the association between key epidemiological factors–age, gender, smoking, hypertension, diabetes, heart disease, chronic obstructive pulmonary disease (COPD), chronic kidney disease (CKD) and chronic liver disease (CLD)–and progression to death and severe disease in patients hospitalized due to COVID-19. We additionally compared the 1) the prevalence of risk factors and death in the US-Europe with that of China; 2) the prevalence of co-morbidities at baseline with the general population prevalence, and 3) prevalence of cardiovascular disease, COPD and CKD at baseline with corresponding organ injuries (acute cardiac injury, acute lung injury, and acute kidney injury) during hospital admission.

## Methods

### Literature search, study selection and data abstraction

We searched Medline, Embase, Web of Science and the WHO COVID-19 database to identify studies published through August 31, 2020 that investigated the risk of severe disease or death in hospitalized patients with confirmed COVID-19 disease. We used search terms, ‘coronavirus disease 19’, ‘COVID-19’, ‘severe acute respiratory syndrome coronavirus 2’ and ‘SARS-CoV-2’ for COVID-19 and the string ((characteristics) OR (risk factors) OR (epidemiology) OR (prevalence) OR (intensive care) OR (ventilator) OR (mechanical ventilator) OR (mortality) OR (survivor*) OR (smoking) OR (smoker*)) AND ((COVID-19) OR (COVID) OR (coronavirus)) for studies published between December 15, 2019 and August 31, 2020. We started the search on March 18, 2020 with biweekly search thereafter and final search on August 31, 2020. We included case series and observational studies that described the prevalence of death or severe disease in adult population stratified by risk factors: age, sex, hypertension, diabetes, heart disease, COPD, CKD and CLD. We excluded studies that included non-consecutive patients or exclusively focused on pregnant women, children, and elderly patients. We excluded studies that exclusively studied critically ill patients from calculation of prevalence of death but included them for calculating the association of risk factors with death. Screening of abstracts and full-text reviews were conducted using Covidence (Melbourne, Australia).

### Risk factors and outcomes

Primary outcomes were prevalence of death and association of risk factors with death. We extracted data on death as recorded in the publications. We measured prevalence of severe disease and association with risk factors as secondary outcomes. We defined outcome as severe disease for any of 1) the study classified COVID-19 disease as severe or critical, 2) intensive care unit (ICU) admission, 3) acute respiratory distress syndrome, or 4) mechanical ventilation. Severe disease was defined by studies as respiratory rate≥30 per minute, oxygen saturation≤93%, and PaO_2_/FiO_2_<300 and/or lung infiltrates>50% within 24–48 hours [3]. Critical illness was defined as respiratory failure, shock and/or multiple organ dysfunction or failure [3]. Heart disease as a pre-existing condition was broadly defined by most studies as ‘cardiovascular disease’ (CVD). Additional outcomes were acute cardiac and kidney injury in the hospitalized patients that were defined as such by the studies.

### Statistical analysis

We calculated and reported summary estimates from fixed-effects models [21]. We assessed heterogeneity across studies using Cochran’s Q-test (*χ*^2^ p value <0.10) [22] and *I*^*2*^ statistics (*I*^*2*^ *>*30%) [23]. In the presence of heterogeneity, we adjusted the confidence intervals for between-study heterogeneity using the method described by Shore et al. [24]. We presented the results from random effects meta-analysis as well. The meta-analysis was performed in Microsoft^®^ Excel 2020 (Microsoft Corporation, Redmond, WA). We analyzed publication bias using funnel plots and Egger’s tests. Quality of each study was assessed using the Newcastle-Ottawa assessment scales using the PRISMA guidelines. We calculated the following as a part of our analyses: 1) prevalence of severe disease or death, 2) prevalence of risk factors, and 3) relative risk for the association of age, sex, and comorbidities with outcome. When not reported or when unadjusted odds ratio was presented, we calculated the relative risk (95% CI) using the frequencies provided. Adjusted estimates were used where available. Case fatality risk (and case severity risk) for a specific risk factor was calculated as number of deaths (or severe disease) in patients with a risk factor out of all patients possessing the risk factor.

## Results

### Study characteristics

**Fig 1:**
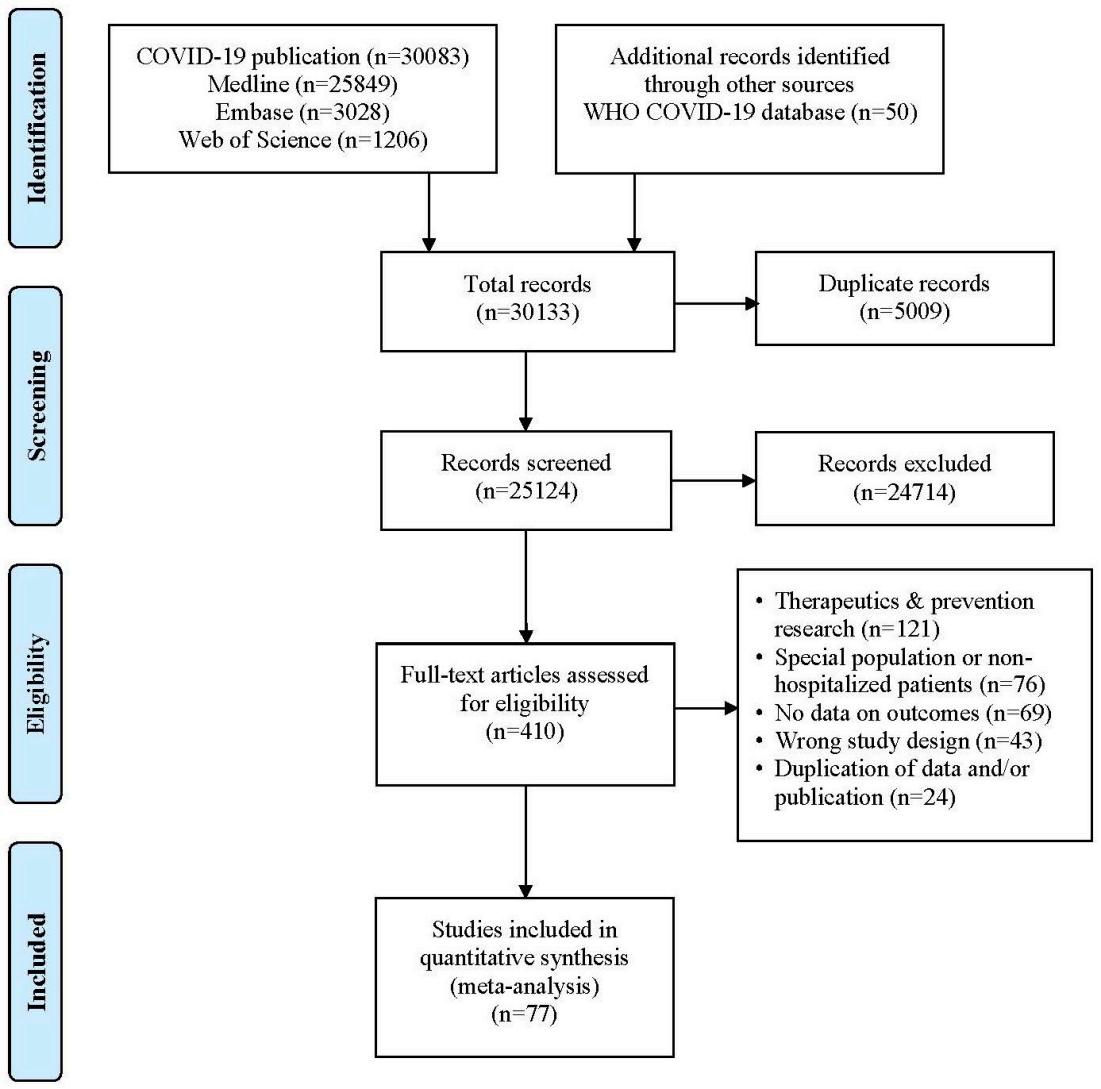
PRISMA flow diagram for selection of studies.

Initial search yielded 30133 citations. Articles were then screened (Fig 1). We identified 410 articles for full text review, of which 77 studies met inclusion criteria (Table 1) [4–8, 13, 14, 25–94]. The studies were conducted in: China (n = 35), USA (n = 18), Europe (n = 10), rest of Asia (n = 5) and Africa (n = 1). Two studies were prospective, five cross-sectional, and remaining retrospective in nature.

**Table 1:** Characteristics of studies to determine prevalence of risk factors and death or severe disease and their associations in patients hospitalized for COVID-19 globally.

Author, year of publication (journal)	Country	Region	Study Period	Study Design	Size	Epidemiological Risk Factor	Outcome	Measures of Association
Aggarwal S et al., 2020 (Diagnosis)	USA	Des Moines	3-1-2020 to 4-4-2020	Retrospective	16	Age, sex, smoking, substance use, obesity, HTN, DM, CVD, COPD, CKD, Cancer	Prevalence of death and primary end point (death, shock, or ICU admission). Association of risk factors with outcome	Unadjusted RR calculated
Argenziano M. G et al., 2020 (BMJ)	USA	New York City	3-11-2020 to 4-6-2020	Retrospective	1,000	Age, sex, ethnicity, obesity, smoking, HTN, DM, CVD, COPD, CKD, cancer, HIV, viral hepatitis, cirrhosis	Association of risk factors with disease severity and death.	Adjusted HR
Brill S. E et al., 2020 (BMC Medicine)	UK	Barnet	3-10-2020 to 4-8-2020	Retrospective	450	Age, race, sex, smoking, HTN, DM, CVD, immunosuppression	Prevalence of death.	Unadjusted RR calculated
							Association of comorbidities with disease severity.	
Cao Z et al., 2020 (PLOS ONE)	China	Beijing	1-21-2020 to 2-12-2020	Retrospective	80	Sex, age, HTN, CVD, DM, COPD, smoking	Association of risk factors with disease severity.	Unadjusted RR calculated
CDC (MMWR)	USA	National	2-12-2020 to 3-28-2020	Retrospective	5285	Age, Current Smoker, DM, CVD, COPD, CKD, CLD	Prevalence of ICU admission. Association of risk factors with severe disease (ICU admission).	Unadjusted RR calculated
Chen G et al., 2020 (Journal of Clinical Investigation)	China	Wuhan	December 2019 to 01-27-2020	Retrospective	21	Age, sex, Huanan sea food market exposure, HTN, DM	Prevalence of severe disease. Compared moderate and severe cases based on risk factors.	Unadjusted RR calculated
Chen J et al., 2020 (Journal of Infection)	China	Shanghai	1-20-2020 to 2-6-2020	Retrospective	249	Age, sex	Prevalence of ICU admission. Association of age and sex with ICU admission.	Adjusted OR reported for age and sex
Chen Q et al., 2020 (Infection)	China	Zhejiang province	1-1-2020 to 3-11-2020	Retrospective	145	Age, sex, smoking, exposure history, BMI, HTN, DM, COPD, CKD, Solid tumor, Heart disease, HIV infection	Prevalence of severe disease. Association of risk factors with severe disease.	Unadjusted RR calculated
Chen T et al., 2020 (BMJ)	China	Wuhan, Hubei	1-13-2020 to 2-28-2020	Retrospective	274	Age, sex, sea food market exposure, contact history, smoking HTN, DM, CVD, CHF, heart failure, cancer, HBV, HIV, CKD	Association of risk factors with death.	Unadjusted RR calculated
							Compared death and recovered group. Presently hospitalized patients excluded from study.	
Chilimuri S et al., 2020 (West J Emerg Med)	USA	New York City	3-9-2020 to 4-9-2020	Retrospective	375	Age, sex, ethnicity, HTN, DM, CVD, COPD, CKD, HIV/AIDS, CLD	Association of risk factors with disease severity and death.	Adjusted OR
								reported for age, sex and comorbidities
Ciceri F et al., 2020 (Clinical Immunology)	Italy	Milan	2-25-2020 to 5-1-2020	Retrospective	410	Age, sex, ethnicity, BMI, HTN, CVD, DM, COPD, CKD, cancer	Prevalence of death.	Adjusted HR
							Association of risk factors with disease severity.	
Cummings MJ et al., 2020 (The Lancet)	USA	New York City	3-2-2020 to 4-1-2020	Prospective	257	Age, sex, race, BMI, HTN, DM, chronic cardiac disease (CHD and CHF), CKD, smoking history, COPD, cancer, HIV, cirrhosis	Association of risk factors with death.	Adjusted HR
Deng Y et al., 2020 (Chin Med J)	China	Wuhan	1-1-2020 to 2-21-2020	Retrospective	116 out of 964	Age, sex, HTN, DM, Heart Disease, Cancer	Association of risk factors with death.	Unadjusted RR calculated
							Compared death and recovered group.	
							Presently hospitalized patients excluded from study.	
Du R-H et al., 2020 (ERJ)	China	Wuhan, Hubei	1-25-2020 to 2-7-2020	Retrospective	179	Age, sex, HTN, DM, CVD, TB, cancer, CKD or CLD	Prevalence of death. Association of risk factors with death.	Adjusted OR for age≥65 and CVD. Unadjusted RR calculated for other variables
Escalera-Antezana et al., 2020(Infez Med)	Bolivia	Nationwide	3-2-2020 to 3-29-2020	Retrospective	107	Age, HTN, CVD, DM, obesity, sex	Prevalence of death.	Adjusted OR
							Association of risk factors with disease severity.	reported for age, sex and risk factors
Feng Y et al., 2020 (AJRCCM)	China	Wuhan, Shanghai, Anhui province	1-1-2020 to 2-15-2020	Retrospective	476	Age, age groups, sex, Wuhan exposure, smoking, alcohol, HTN, anti-hypertensives, CVD, DM, cancer, COPD, CKD	Prevalence of death. Association of risk factors with severe disease.	Adjusted HR for HTN, CVD, DM. Unadjusted RR calculated for other variables
Ferguson J et al., 2020 (EID)	USA	Northern California	03-13-2020 to 04-11-2020	Retrospective	72	Sex, race, smoking, HTN, DM, CKD, Heart Disease, COPD	Prevalence of ICU admission. Association of risk factors with severe disease (ICU admission).	Unadjusted RR calculated
Galloway J.B et al., 2020 (Journal of Infection)	UK	London	3-1-2020 to 4-17-2020	Retrospective	1,157	Age, sex, ethnicity, cancer, CKD, DM, HTN, CVD, COPD	Prevalence of death.	Adjusted HR reported for age and sex
							Association of risk factors with disease severity.	
Garibaldi B et al., 2020 (Ann Intern Med)	USA	Maryland	3-4-2020 to 6-27-2020	Retrospective	832	Age, sex, alcohol, smoking, BMI, cancer, CVD, COPD, HTN, liver disease, CKD, HIV/AIDS DM	Association of risk factors with disease severity.	Adjusted HR
		Washington DC						
								reported for age, ethnicity and BMI
Giacomelli A et al., 2020 (Pharmacol Res)	Italy	Milan	2-21-2020 to 4-20-2020	Prospective	233	Sex, age, smoking, obesity	Prevalence of death.	Adjusted HR
							Association of risk factors with disease severity.	reported for sex, age, and obesity
Gold J et al, 2020 (MMWR)	USA	Georgia	3-1-2020 to 3-30-2020	Retrospective	305	Age, sex, race, HTN, DM, Heart Disease, COPD, CKD, Cancer	Prevalence of patient characteristics, death, and ICU.	Unadjusted RR calculated
Goyal P et al. 2020 (NEJM)	USA	New York City	3-3-2020 to 3-27-2020	Retrospective	393	Age, sex, race, smoking, HTN, DM, COPD, Heart Disease, Asthma	Prevalence of severe disease (mechanical ventilation). Association of risk factors with severe disease.	Unadjusted RR calculated
Gregoriano C et al.,2020 (Swiss Medical Weekly)	Switzerland	Aarau	2-26-2020 to 4-30-2020	Retrospective	99	Age, sex, smoking, HTN, cancer, CVD, COPD, obesity, DM, rheumatic disease, organ transplant recipient, obstructive sleep apnea	Prevalence of disease endpoints (transfer to ICU and in-hospital mortalities).	Unadjusted OR
							Association of comorbidities with disease endpoints.	
Guan et al., 2020 (NEJM)	China	Nationwide	12-11-2019 to 01-31-2020	Retrospective	1099	Age, sex, smoking, exposure to transmission source, HTN, DM, CHD, CKD, COPD, Cancer, HBV, cerebrovascular disease, immunodeficiency	Prevalence of death, composite outcome, ((Death/MV/ICU) and severe disease. Association with severe disease and composite outcome.	Unadjusted RR calculated
Guan Wei-Jie, 2020(ERJ)	China	Nationwide	12-11-2019 to 1-31-2020	Retrospective	1590	Age, sex, smoking, CKD, COPD, HTN, DM, CVD, Cancer, HBV	Prevalence of patient characteristics, death and composite outcome (Death, ICU, MV).	Adjusted HR
Hewitt J et al., 2020 (Lancet)	UK	Nationwide (UK),	2-27-2020 to 4-28-2020	Prospective	1,564	Age, sex, smoking, DM, HTN, CVD, CKD	Prevalence of death.	Adjusted HR
	Italy	Modena (Italy)					Association of risk factors with disease severity.	
Hsu H. E et al., 2020 (Morbidity and Mortality Weekly Report)	USA	Boston	3-1-2020 to 5-18-2020	Retrospective	2,729	Age, sex, ethnicity, COPD, cancer, CKD, cirrhosis, CVD, DM, HIV/AIDS, HTN, obesity, substance use disorder	Association of risk factors with disease severity.	Unadjusted RR calculated
Hu L et al., 2020 (CID)	China	Wuhan	1-8-2020 to 2-20-2020	Retrospective	323	Age, sex, current smoker, HTN, DM, CVD, COPD, CKD, CLD, Cancer	Prevalence of severe (severe and critical) disease. Association of risk factors with disease severity.	Unadjusted RR calculated
Huang C et al., 2020 (The Lancet)	China	Wuhan	12-16-2020 to 1-2-2020	Prospective	41	Age, sex, Huanan seafood market exposure, smoking, HTN, DM, CKD, COPD, CVD, Cancer, CLD	Association of risk factors with severe disease (ICU care).	Unadjusted RR calculated
Hur K et al., 2020 (Otolaryngol Head Neck Surg)	USA	Chicago	3-1-2020 to 4-8-2020	Retrospective	486	Age, sex, BMI, smoking, HTN, DM, CVD, COPD, cancer, immunosuppression, CKD,	Association of risk factors with disease severity.	Adjusted HR (for age, sex, ethnicity BMI, HTN, smoking)
Iaccarino G et al., 2020 (Hypertension)	Italy	Nationwide	3-9-2020 to 4-9-2020	Cross-sectional	1,591	Age, sex, HTN, obesity, DM, COPD, CKD, CVD, cancer	Prevalence of death.	Adjusted OR
							Association of risk factors with disease severity.	
Inciardi R et el., 2020 (Eur Heart J)	Italy	Lombardy	3-4-2020 to 3-25-2020	Retrospective	99	Sex, smoking, HTN, DM, coronary artery disease, COPD, CKD, cancer	Prevalence of death. Association of risk factors with death.	Unadjusted RR calculated
Jang J.G et al., 2020 (Journal of Korean Medical Science)	South Korea	Daegu	2-19-2020 to 4-15-2020	Retrospective	110	Age, sex, CVD, cerebrovascular disease, COPD, dementia, DM, HTN, connective tissue disease liver disease, malignancy, Parkinson’s disease	Association of risk factors with disease severity and death.	Adjusted OR
Javanian M et al., 2020 (Rom J Intern Med)	Iran	Mazandaran province	2-25-2020 to 3-12-2020	Retrospective	100	Age, sex, HTN, DM, CVD, CKD, cancer, CLD	Prevalence of death. Association of risk factors with death.	Unadjusted RR calculated
Kalligeros M et al., 2020 (Obesity Journal)	USA	Rhode Island	2-17-2020 to 4-5-2020	Retrospective	103	Age, sex, ethnicity, smoking, BMI (obesity), cancer, CKD, cirrhosis, DM, heart disease (CVD), HTN, lung disease (COPD), transplant	Association of risk factors with disease severity.	Adjusted OR
								(for age, sex, ethnicity, BMI, DM, HTN, heart disease, lung disease)
Khalil K et al., 2020 (Journal of Infection)	UK	London	3-7-2020 to 4-7-2020	Prospective	220	Age, sex, ethnicity, smoking, COPD, CVD, HTN, hyperlipidemia, DM, CKD, CVA, dementia, liver disease, cancer	Prevalence of death.	Unadjusted RR calculated
							Association of risk factors with disease severity.	
Khamis F et al., 2020 (Journal of Infection and Public Health)	Oman	Muscat	2-24- 2020 to 4-24-2020	Retrospective	63	Age, sex, smoking, substance use, HTN, DM, CKD, CVD	Prevalence of severe disease and death.	Unadjusted RR calculated
							Association of risk factors with disease severity.	
Lendorf M.E et al., 2020 (Danish Medical Journal)	Denmark	North Zealand	3-1-2020 to 5-18-2020	Retrospective	111	Age, sex, BMI, cancer, HTN, CVD, COPD, immunosuppression, CKD, DM, smoking	Association of risk factors with disease severity and death.	Unadjusted RR calculated
Li X et al., 2020 (J Allergy Clin Immunol)	China (Wuhan, Hubei)	Wuhan, Hubei	1-26-2020 to 2-5-2020	Retrospective	548	Age, sex, smoking, HTN, DM, Heart Disease, CKD, Cancer, COPD	Prevalence of death and severe disease.	Unadjusted RR calculated
							Association of risk factors with severe disease.	
Liu S et al., 2020 (BMC Infectious Diseases)	China	Jiangsu Province	1-10-2020 to 3-15-2020	Retrospective	625	Sex, age, HTN, DM, CVD	Association of risk factors with disease severity.	Adjusted OR
								(for age and HTN)
Liu W et al. 2020 (Chin Med J)	China	Wuhan	12-30-2019 to 01-15-2020	Retrospective	78	Age, sex, smoking history, exposure to Huanan seafood market, HTN, diabetes, COPD, cancer	Compared progression group and stabilization group. Progression group defined by progression to severe or critical disease or death.	Unadjusted RR calculated
Nikpouraghdam M et al., 2020 (J Clin Virol)	Iran	Tehran	2-19-2020 to 4-15-2020	Retrospective	2,964	Age, sex, DM, COPD, HTN, CVD, CKD, cancer	Prevalence of death.	Adjusted OR
							Association of risk factors with disease severity.	
Nowak B et al., 2020 (Pol Arch Intern Med)	Poland	Warsaw	3-16-2020 to 4-7-2020	Retrospective	169	Sex, smoking, HTN, DM, CVD, COPD, CKD, AKI, cancer	Prevalence of death. Association of risk factors with death.	Unadjusted RR calculated
Okoh A.K et al., 2020 (Int J Equity Health)	USA	Newark	3-10-2020 to 4-20-2020	Retrospective	251	Age, sex, ethnicity, BMI, HTN, DM, CVD, COPD, HIV, CKD, cancer	Prevalence of death.	Adjusted OR
							Association of risk factors with disease severity and death.	
Palaiodimos L et al., 2020 (Metabolism)	USA	New York	3-9-2020 to 3-22-2020	Retrospective	200	Age, sex, race, smoking, HTN, DM, coronary artery disease, COPD, CKD, cancer	Prevalence of death. Association of risk factors with death.	Adjusted OR (provided by the study)
Pellaud C et al., 2020 (Swiss Medical Weekly)	Switzerland	Fribourg	3-1-2020 to 5-10-2020	Retrospective	196	Sex, age, HTN, DM, obesity, CVD, COPD, cancer, immunosuppression, smoking	Prevalence of death.	Unadjusted RR calculated
							Association of risk factors with disease severity.	
Richardson S et al., 2020 (JAMA)	USA	New York	3-1-2020 to 4-4-2020	Retrospective	5700	Age, sex, race, smoking, HTN, DM, COPD, asthma, coronary artery disease, kidney disease, liver disease, obesity, cancer	Prevalence of ICU admission and death.	Unadjusted RR calculated
							Association of risk factors with death.	
Rivera-Izquierdo M et al., 2020 (PLOS ONE)	Spain	Granada	3-16-2020 to 4-10-2020	Retrospective	238	Sex, age, smoking, HTN, DM, CVD, COPD, CKD, active neoplasia, medications	Prevalence of death.	Adjusted HR
							Association of risk factors with disease severity.	
Shabrawishi M et al., 2020 (Plos One)	Saudi Arabia	Mecca	3-12-2020 to 4-8-2020	Retrospective	150	Age, sex, HTN, DM, CVD, CKD, hypothyroidism, cancer, CVA, COPD, CLD	Association of risk factors with disease severity and death.	Unadjusted RR calculated
Shahriarirad R et al., 2020 (BMC Infectious Diseases)	Iran	Fars Province	2-20-2020 to 3-20-2020	Multicenter Retrospective	113	Age, sex, HTN, DM, CVD, COPD, CKD, malignancy, other immunosuppressive diseases	Prevalence of death.	Unadjusted RR calculated
							Association of risk factors with disease severity.	
Shekhar R et al., 2020 (Infectious Diseases)	USA	New Mexico	1-19-2020 to 4-24-2020	Cohort	50	Age, sex, HTN, DM, COPD, alcoholic cirrhosis, alcohol use, obesity	Association of risk factors with disease severity.	Unadjusted RR calculated
Shi Y et al., 2020 (Crit Care)	China	Zhejiang province	Not specified to 02-11-2020	Retrospective	487	Age, sex, smoking, HTN, DM, CKD, CVD, CLD, cancer	Prevalence of and association of risk factors with severe disease	Unadjusted RR calculated
Suleyman G et al., 2020 (JAMA Network)	USA	Metropolitan Detroit	3-9-2020 to 3-27-2020	Retrospective	463	Age, sex, ethnicity, COPD, obstructive sleep apnea, DM, HTN, CVD, CKD, cancer, rheumatologic disease, organ transplant, obesity, smoking	Association of risk factors with disease severity.	Adjusted OR
Sun L et al., 2020 (Journal of Medical Virology)	China	Beijing	1-20-2020 to 2-15-2020	Retrospective	55	Age, sex, exposure, HTN, DM, CVD, Lung Disease, CKD, CLD	Prevalence of severe disease. Association of risk factors with severe disease.	Unadjusted RR calculated
Tambe M et al., 2020 (Indian J Public Health)	India	Pune	3-31-2020 to 4-24-2020	Cross-Sectional	197	Age, sex, HTN, DM, COPD, CVS, ALD, CKD	Association of risk factors with disease severity and death.	Unadjusted RR calculated
Tian S et al., 2020 (Journal of Infection)	China	Beijing	1-20-2020 to 2-10-2020	Retrospective	262	Age, sex, contact history, exposure to Wuhan.	Prevalence of death. Association of severe disease with risk factors.	Unadjusted RR calculated
Tomlins J et al., 2020 (Journal of Infection)	UK	Bristol	3-10-2020 to 3-30-2020	Retrospective	95	Age, sex, HTN, DM, COPD, CVD, cancer, renal disease, gastrointestinal disease, neurological disease	Prevalence of death. Association of risk factors with death.	Unadjusted RR calculated
Turcotte J.J et al., 2020 (PLOS ONE)	USA	Maryland	3-1-2020 to 4-12-2020	Retrospective	117	Age, BMI, sex, DM, obstructive sleep apnea, COPD, CVD, CKD, HTN, smoking, alcohol use, liver disease	Association of risk factors with disease severity and death.	Adjusted OR
Wan S et al., 2020 (Journal of Medical Virology)	China	Northeast Chongqing	1-23-2020 to 2-8-2020	Retrospective	135	Age, sex, smoking, CKD, COPD, HTN, DM, CVD, Cancer, CLD, exposure, travel history	Prevalence of severe disease. Association of risk factors with severe disease.	Unadjusted RR calculated
Wang D et al., 2020 (JAMA)	China	Wuhan	1-1-2020 to 1-28-2020	Retrospective	138	Age, sex, Huanan Seafood Market Exposure, HTN, DM, CVD, COPD, Cancer, CKD, CLD, HIV	Prevalence of death and ICU admission.	Unadjusted RR calculated
							Association of risk factors with severe disease (ICU care)	
Wang R et al., 2020 (Internal Journal of Infectious Diseases)	China	Fuyang	1-20-2020 to 02-09-2020	Retrospective	125	Age, sex, CVD, Cancer	Prevalence of critical disease. Association of age, sex, and smoking with critical disease.	Unadjusted RR calculated
Wang Z et al., 2020 (CID)	China	Wuhan	1-16-2020 to 01-29-2020	Retrospective	69	Age, sex, HTN, DM, CVD, COPD, Cancer, HBV, Asthma	Prevalence of death and severe disease (SpO2<90%). Association of risk factors with severe disease.	Unadjusted RR calculated
Wei Y et al., 2020 (BMC Infectious Diseases)	China	Hubei Province	1-27-2020 to 3-22-2020	Retrospective	276	Age, sex, smoking, obesity, HTN, COPD, CVD, DM, cerebrovascular disease, cancer	Association of risk factors with disease severity.	Unadjusted RR calculated
Wu C et al., 2020 (JAMA Intern Med)	China	Wuhan	12-15-2019 to 01-26-2020	Retrospective	201	Age, sex, HTN, DM, CVD, CKD, Chronic Lung Disease, Cancer, CLD, Sea Food Market Exposure.	Prevalence of ARDS, ICU admission and death. Association of risk factors with severe disease (ARDS) and death.	Unadjusted RR calculated
Yang X et al, 2020 (Lancet Respir Med)	China	Wuhan	12-24-2019 to 1-26-2020	Retrospective	52	Age, sex, exposure, COPD, diabetes, chronic cardiac disease, smoking, malnutrition	Association of risk factors with death.	Unadjusted RR calculated
Yao Q et al., 2020 (Pol Arch Intern)	China	Huanggang, Hubei	1-30-2020 to 2-11-2020	Retrospective	108	Age, sex, smoking, HTN, DM, CVD, CLD, cancer	Prevalence of severe disease and death.	Unadjusted RR calculated
							Association of risk factors with severe disease and death.	
Young BE et al., 2020 (JAMA)	Singapore	Singapore	1-23-2020 to 2-3-2020	Retrospective	18	Age, sex	Prevalence of severe disease (receiving supplemental O2). Association of severe disease with age and sex.	Unadjusted RR calculated
Yu T et al., 2020 (Clinical Therapeutics)	China	Guangdong	January to February 2020	Cross-sectional	95	Age, sex, current smoker	Prevalence of ARDS.	Unadjusted RR calculated
							Association of age, sex, and smoking with ARDS.	
Yu X et al., 2020 (Transboundary and Emerging Diseases)	China	Shanghai	Up to 2-19-2020	Retrospective	333	Age, sex, BMI, smoking, alcohol, exposure, HTN, DM, CVD	Prevalence of death and severe disease (Severe/critical pneumonia). Association of risk factors with severe disease.	Adjusted OR for age group, sex, CVD, DM, HTN.
Zhan T et al., 2020 (J Int Med Res)	China	Wuhan	1-12-2020 to 3-8-2020	Retrospective	405	Age, sex, smoking, alcohol history, CVD, gastrointestinal disease, COPD, CKD, CLD	Association of risk factors with disease severity.	Unadjusted RR calculated
Zhang G et al., 2020 BMC Respiratory Research)	China	Wuhan	1-16-2020 to 2-25-2020	Retrospective	95	Age, sex	Prevalence of severe disease, composite end point, and death. Association with severe disease.	Unadjusted RR calculated
Zhang J et al., 2020 (Clin Microbiol Infect)	China	Wuhan	1-11-2020 to 2-6-2020	Retrospective	663	Age, sex, COPD, CVD, gastrointestinal disease, CKD, cancer	Prevalence of death.	Adjusted OR
							Association of risk factors with disease severity.	
Zhang JJ et al., 2020 (Allergy)	China	Wuhan	1-16-2020 to 2-3-2020	Retrospective	140	Age, sex, current smoker, past smoker, exposure history, HTN, DM, CVD, COPD, CKD, CLD	Prevalence of severe disease. Association of risk factors with severe disease (ICU admission).	Unadjusted RR calculated
Zhao X-Y et al., 2020 (BMC Inf Dis)	China	Hubei (Non-Wuhan)	1-16-2020 to 2-10-2020	Retrospective	91	Age, sex, DM, COPD, Cancer, Kidney disease	Prevalence of death. Association of risk factors with severe disease	Unadjusted RR calculated
Zheng S et al., 2020 (BMJ)	China	Zhejiang province	1-19-2020 to 2-15-2020	Retrospective	96	Age, sex, HTN, DM, CVD, lung disease, Liver disease, renal disease, malignancy, viral Load, immunocompromise	Prevalence of death and severe disease.	Unadjusted RR calculated
							Association of risk factors with severe disease.	
Zheng Y et al., 2020 (Pharmacological Research)	China	Shiyan, Hubei	1-16-2020 to 2-4-2020	Retrospective	73	Age, sex, exposure, smoking history, DM, CVD	Prevalence of severe (severe/ critical) disease. Association of smoking and diabetes with severe disease.	Unadjusted RR calculated
Zhou F et al., 2020 (The Lancet)	China	Wuhan	12-29-2019 to 1-31-2020	Retrospective	191	Age, sex, current smoking, exposure history, HTN, DM, CVD, COPD, cancer, CKD	Prevalence of severe disease (ICU admission) and death. Association of risk factors with death.	Adjusted OR for age and CVD. Unadjusted RR calculated for other variables.

CVD, cardiovascular disease; CKD, chronic kidney disease; CLD, chronic liver disease; COPD, chronic obstructive pulmonary disease; HTN, hypertension; DM, diabetes mellitus; ICU, intensive care unit; BMI, body mass index; HIV, human immunodeficiency virus; AIDS, acquired immunodeficiency syndrome; RR, relative risk; HR, hazard ratio; OR, odds ratio.

### Population and demographics

There were 38,906 total COVID-19 hospitalized patients including 21468 patients from the US and Europe (87% from the US), and 9740 patients from China. Median age was 59 years [IQR: 57–62 years; I^2^ = 58%; n = 62 studies] and 48% [95% CI: 44–53%; I^2^ = 98%; n = 41] were aged≥60. Fifty-nine percent [95% CI: 57–60%; I^2^ = 98%; n = 75] of the patients were males.

### Prevalence of death and severe disease

We calculated an overall prevalence of death of 20% [95% CI: 18–23%; I^2^ = 96%; n = 60], ranging from 1% to 38% across the studies, and of severe disease of 28% [95% CI: 24–33%; I^2^ = 98%; n = 60] for all patients hospitalized due to COVID-19 (Tables 2 and 3). Data on prevalence of death, severe disease, and risk factors (S1 Table), and association of the risk factors with death (S2 Table) and severe disease (S3 Table) for the individual studies are presented in the supplemental tables.

**Table 2:** Pooled prevalence of death stratified by epidemiological risk factors in COVID-19 patients hospitalized between December 2019-August 2020.

Risk factor or Outcome	Overall prevalence of risk across studies		Pooled Prevalence of Death (Case Fatality Risk) and Risk Factor			Summary Relative Risk of Death			
	No. of studies	Pooled prevalence of risk factor and death,	No. of studies	*Case fatality risk (Prevalence of death in risk group),	^#^Prevalence of risk factor in persons who died,	No. of studies	Fixed Effects	Random Effects^#^	Heterogeneity
							Summary relative risk; 95% CI (Shore adjusted)	sRR; (95% CI)	*I*^*2*^; c^2^; p value
		% (95% CI)		% (95% CI)	% (95% CI)				
Death	60	20 (18–23)	N/A	N/A	N/A	N/A	N/A	N/A	N/A
Age ≥ 60 years	41	48 (44–53)	18	35 (28–43)	85 (80–89)	24	3.61 (2.96–4.39)	1.29 (1.03–1.62)	77%; 99; p<0.01
Male	75	59 (57–60)	31	26 (21–32)	66 (64–69)	36	1.31 (1.22–1.40)	1.34 (1.24–1.45)	18%; 43; p = 0.17
Smoking history	41	26 (22–31)	11	27 (24–32)	44 (38–50)	13	1.28 (1.06–1.55)	1.41 (1.12–1.78)	68%; 37; p<0.01
Current smoker	21	10 (7–13)	7	21 (14–29)	13 (7–24)	8	1.43 (91–2.26)	1.53 (95–2.45)	78%; 32; p<0.01
COPD	52	9 (8–11)	20	51 (36–71)	12 (7–19)	22	1.70 (1.42–2.04)	1.74 (1.43–2.13)	66%; 61; p<0.01
Hypertension	64	50 (46–54)	29	28 (23–36)	66 (61–70)	32	1.76 (1.58–1.96)	1.88 (1.66–2.13)	56%; 70; p<0.01
Diabetes	67	28 (25–31)	29	24 (17–33)	39 (35–44)	33	1.50 (1.35–1.66)	1.60 (1.42–1.79)	58%; 77; p<0.01
Cardiovascular disease	65	17 (15–20)	29	52 (46–60)	37 (32–43)	34	2.08 (1.81–2.39)	2.25 (1.92–2.64)	69%; 106; p<0.01
Chronic kidney disease	47	13 (11–16)	18	48 (37–63)	27 (21–34)	23	2.52 (2.11–3.00)	2.39 (1.91–2.99)	72%; 79; p<0.01
Chronic Liver Disease	31	2(2–3)	8	39(31–50)	6 (4–8)	9	2.65(1.88–3.75)	1.99 (1.26–3.16)	77%; 35; p<0.01

*Case fatality risk of represent total number of people that died in the specific risk group divided by total population in the risk group.

^#^ Prevalence of risk group in dead represent total number of people having the risk group divided by total population that died.

**Table 3:** Pooled prevalence of severe disease stratified by epidemiological risk factors in COVID-19 patients.

Risk group or outcome	Prevalence of Severe Disease (Case Severity Risk) and Risk Factors			Summary Relative Risk of Severe Disease			
	No. of studies	Prevalence of severe disease and case severity risk*, % (95% CI)	*Prevalence of risk factor in people with severe disease, % (95% CI)	No. of studies	Fixed Effects	Random Effects^#^	Heterogeneity
					sRR; 95% CI (Shore adjusted)	sRR; (95% CI)	*I*^*2*^; c^2^; p value
Severe disease	25	20 (16–25)	N/A	N/A	N/A	N/A	N/A
Age ≥ 60 years	26	48 (39–59)	56 (52–61)	29	1.57 (1.36–1.80)	1.76 (1.50–2.07)	85%; 184; p<0.01
Male	45	40 (34–47)	63 (61–66)	47	1.26 (1.18–1.35)	1.33 (1.22–1.44)	38%; 75; p<0.01
Smoking history	27	39 (34–46)	26 (21–32)	27	1.29 (1.18–1.42)	1.32 (1.18–1.47)	33%; 39; p = 0.05
Current smoker	13	38 (28–53)	13 (9–20)	15	1.52 (1.21–1.91)	1.25 (94–1.66)	75%;56; p<0.01
COPD	24	43 (35–52)	14 (12–17)	29	1.71 (1.49–1.97)	1.83 (1.54–2.18)	84%;179; p<0.01
Hypertension	39	44 (37–53)	55 (50–61)	40	1.46 (1.28,1.65)	1.54 (1.33,1.78)	77%;168; p<0.01
Diabetes	43	43 (38–49)	33 (30–38)	44	1.48 (1.35–1.63)	1.64 (1.47–1.82)	59%;104; p<0.01
Cardiovascular disease	37	56 (48–65)	28 (24–33)	38	1.54 (1.39–1.72)	1.74 (1.52–1.98)	77%;164; p<0.01
Chronic kidney disease	22	36 (33–40)	26 (19–37)	27	1.56 (1.31–1.86)	1.42 (1.15–1.76)	85%; 176; p<0.01
Chronic Liver Disease	12	43(32–57)	5 (3–7)	15	1.63 (1.23–2.15)	1.66 (1.16–2.36)	82%; 76; p<0.01

*Case severity risk represent total number of people developing severe disease in the specific risk group divided by total population in that risk group.

^#^ Prevalence of risk factor in severe disease represent total number of people with the risk factor divided by total population with severe disease.

### Predictors of death and severe disease (Tables 2 and 3)

#### Age and sex

Median age for people who died was 79 years [IQR: 77–80; I^2^ = 89%; n = 28] and who had severe disease was 61 years [IQR: 59–63; I^2^ = 48%; n = 26]. Eighty-five percent [95% CI: 80–89; I2 = 76%; n = 18] of the deaths were in people aged ≥ 60 years and 66% [95% CI: 64–69; n = 34] were in males. The CFR (95% CI) was 35% (28–43%) for age≥60 years and 26% (21–32%) for males. Patients aged≥60 years [summary relative risk (sRR): 3.61; 95% CI: 2.96–4.39; I^2^ = 77%; n = 24] and males [sRR: 1.34; 95% CI: 1.22–1.40; I^2^ = 18%; n = 36] had higher risk of death. The risk of severe disease was similarly higher for age>60 [sRR: 1.57; 95% CI: 1.36–1.80; I^2^ = 85%; n = 29] and males [sRR: 1.26; 95% CI: 1.18–1.35; I^2^ = 38%; n = 47].

#### Hypertension

**Fig 2:**
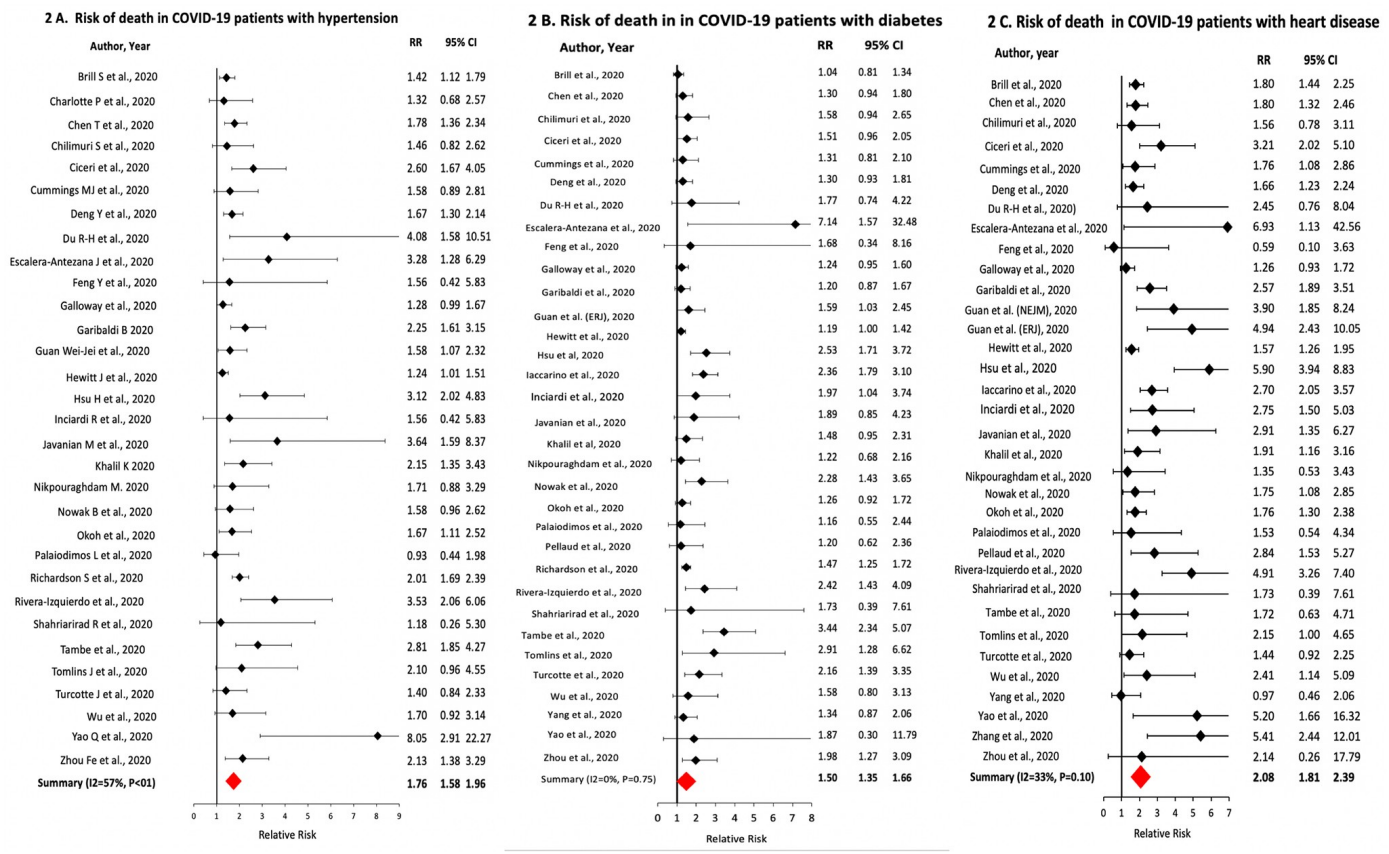
Association of hypertension, diabetes and heart disease with death in COVID-19 patients.

The prevalence of hypertension in the COVID-19 patients was 50% [95% CI: 46–54% I^2^ = 98%; n = 64], with a CFR in hypertensive patients of 28% [95% CI: 23–36%; I^2^ = 97%; n = 29] and a CSR of 44% [95% CI: 37–53%; I^2^ = 95%; n = 39]. Of the COVID-19 patients that died, 66% [95% CI: 61–70%; I^2^ = 83%; n = 29] had hypertension. Hypertensives had higher relative risk of death [sRR: 1.76; 95% CI: 1.58–1.96; I^2^ = 56%; n = 32] and severe disease [sRR: 1.46; 95% CI: 1.28–1.65; I^2^ = 77%; n = 40] compared to non-hypertensives (Fig 2A).

#### Diabetes

The prevalence of diabetes was 28% [95% CI: 25–31%; I^2^ = 97%; n = 67] with a CFR of 24% [95% CI: 17–33%; I^2^ = 98%; n = 29] and CSR of 43% [95% CI: 38–49%; I^2^ = 99%; n = 30] in the diabetics. Of the COVID-19 patients that died, 33% [95% CI: 32–44%; I^2^ = 83%; n = 29] were diabetics. Diabetics had higher relative risk of death [sRR: 1.50; 95% CI: 1.35–1.66; I^2^ = 58%; n = 33] and severe disease [sRR: 1.48; 95% CI: 1.35–1.63; I^2^ = 59%; n = 44] compared to non-diabetics (Fig 2B).

#### Cardiovascular disease

The pooled prevalence of CVD was 17% [95% CI: 15–20%; I^2^ = 96%; n = 65] with a CFR of 52% [95% CI: 46–60%; I^2^ = 81%; n = 29] and CSR of 56% [95% CI: 48–65%; I^2^ = 91%; n = 37] among cardiac patients. Of the patients that died, 37% [95% CI: 32–43%; I^2^ = 83%; n = 29] had CVD. Patients with CVD had higher relative risk of death [sRR: 2.08; 95% CI: 1.81–2.39; I^2^ = 69%; n = 34] and severe disease [sRR: 1.54; 95% CI: 1.39–1.72; I^2^ = 77%; n = 38] compared to patients without CVD (Fig 2C).

#### Smoking and COPD

**Fig 3:**
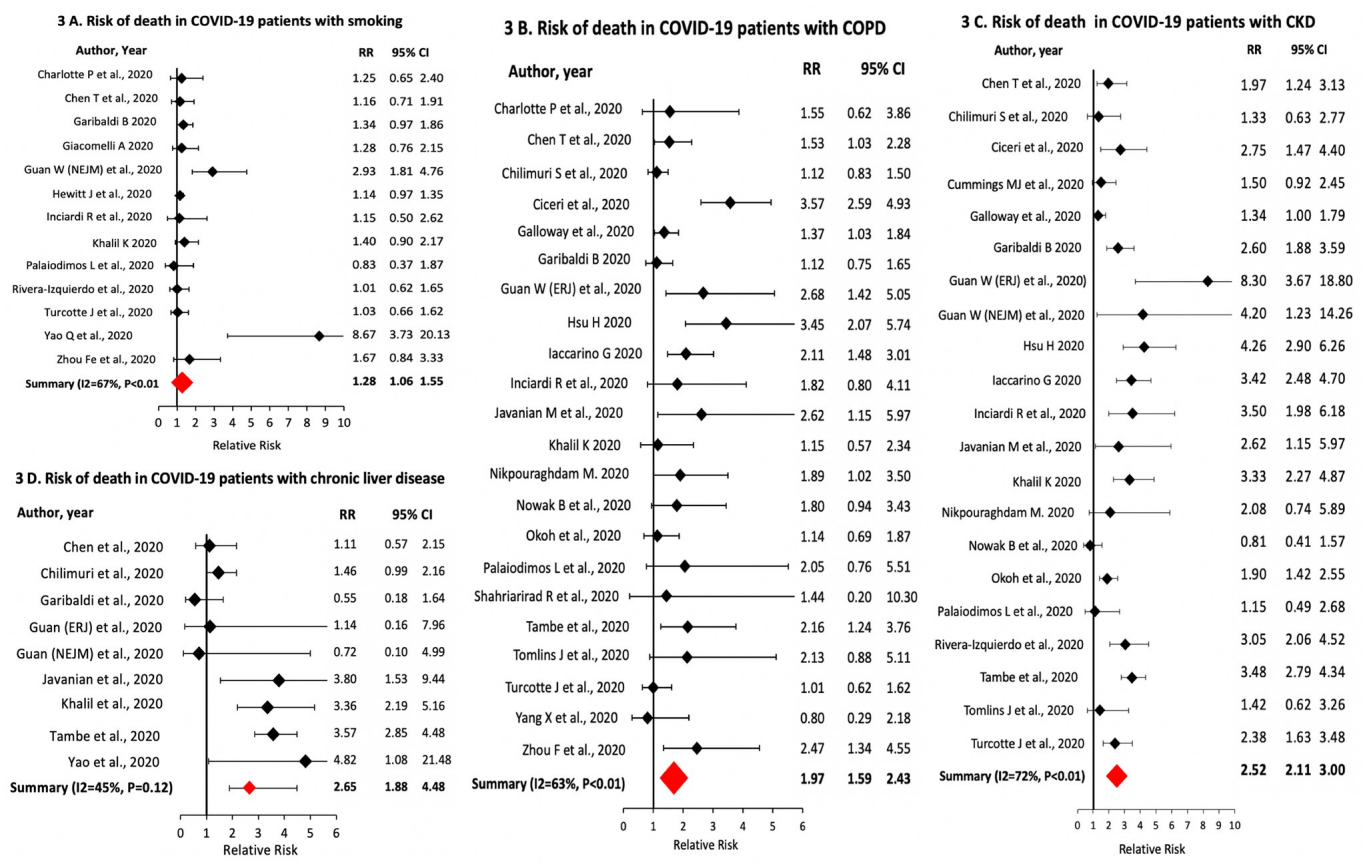
Association of smoking, chronic obstructive pulmonary disease, chronic kidney disease and chronic liver disease with death in COVID-19 patients.

The prevalence of any history of smoking in the patients was 26% [95% CI: 22–31%; I^2^ = 98%; n = 41]. For patients with smoking history, the CFR was 27% [95% CI: 24–32%; I^2^ = 61%; n = 11] and CSR was 39% [95% CI: 34–46; I^2^ = 78%; n = 27]. Compared to never smokers, patients with smoking history had higher relative risk of death [sRR: 1.28; 95% CI: 1.06–1.55; I^2^ = 68%; n = 13] and severe COVID-19 disease [sRR: 1.29; 95% CI: 1.18–1.42; I^2^ = 33%; n = 27] (Fig 3A). The prevalence of COPD was 9% [95% CI: 8–11%; I^2^ = 94%; n = 52]. Patients with COPD had a CFR of 51% [95% CI: 43–59%; I^2^ = 0%; n = 21]; CSR of 43% [95% CI: 35–52%; I^2^ = 84%; n = 24]; a sRR of death of 1.70 [95% CI: 1.42–2.04; I^2^ = 66%; n = 22] and of severe disease of 1.71 [95% CI: 1.49–1.97; I^2^ = 84%; n = 29] (Fig 3B).

#### Chronic kidney disease

The prevalence of CKD was 13% [95% CI: 11–16%; I^2^ = 96%; n = 47] with a CFR of 48% [95% CI: 37–63%; I^2^ = 89%; n = 18] and CSR of 36% [95% CI: 33–40%; I^2^ = 56%; n = 22] in CKD patients. CKD was present in 27% [95% CI: 21–34%; I^2^ = 79%; n = 18] of all COVID-19 patients that died. CKD patients had higher relative risk of death [sRR: 2.52; 95% CI: 2.11–3.00; I^2^ = 72%; n = 23] and severe disease [sRR: 1.56; 95% CI: 1.31–1.86; I^2^ = 85%; n = 27] compared to non-CKD patients (Fig 3C).

#### Chronic liver disease

The prevalence of CLD was 2% [95% CI: 2–3%; I^2^ = 72%; n = 31] with a CFR of 39% [95% CI: 31–50%; I^2^ = 0%; n = 8] and CSR of 43% [95% CI: 32–57%; I^2^ = 5%; n = 12] in CLD patients. CLD was present in 6% [95% CI: 4–8%; I^2^ = 0%; n = 8] of the COVID-19 patients who died. Patients with CLD had higher relative risk of death [sRR: 2.65; 95% CI: 1.88–3.75; I^2^ = 77%; n = 9] and severe disease [sRR: 1.63; 95% CI: 1.23–2.15; I^2^ = 82%; n = 15] compared to non-CKD patients (Fig 3D).

### COVID-19 related organ system injury

**Fig 4:**
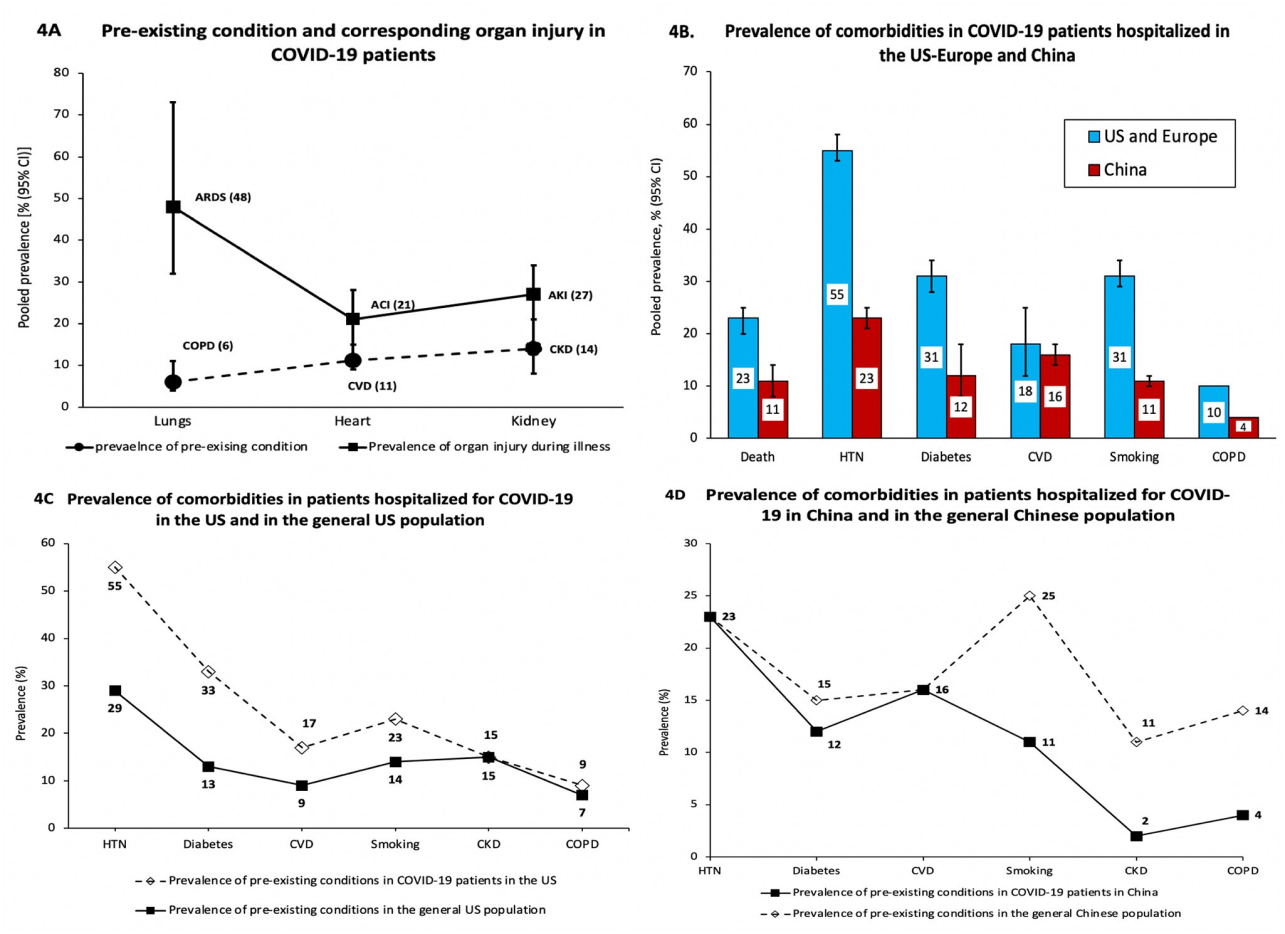
Prevalence of acute organ injuries during hospital stay and regional difference in prevalence of death and comorbidities in patients hospitalized for COVID-19. ARDS, acute respiratory distress syndrome; COPD, chronic obstructive pulmonary disease; ACI, acute cardiac injury; CVD, cardiovascular disease; AKI, acute kidney injury; CKD, chronic kidney disease; HTN, hypertension.

To understand how pre-existing health conditions may be correlated with the risk of specific organ injury, we calculated the prevalence of acute injury to lung, heart and kidney for studies that reported prevalence of both the pre-existing condition(s) and corresponding organ injury (Fig 4A). Pooled across 12 studies [14, 25, 32, 45, 48, 49, 52, 54, 60, 62, 79], the prevalence of COPD at baseline was 6% [95% CI: 4–11%] and the proportion of patients developing ARDS during hospitalization was 48% [32–73%]. The pooled prevalence of baseline CVD (n = 13 studies) was 11% [95% CI: 9–15%] and that of acute cardiac injury (ACI) during hospitalization was 21% [95% CI: 15–28%] [6, 14, 25, 32, 35, 43, 48, 49, 54, 79, 84]. The prevalence of CKD (n = 12 studies) was 14% [95% CI: 8–26%] and that of acute kidney injury during hospitalization (AKI) was 27% [95% CI: 21–34%] [6, 14, 25, 32, 45, 48, 65, 79].

### Regional difference in prevalence of death and risk factors

Upon sub-group analysis, we noted significantly higher prevalence of death and risk factors among COVID-19 patients in the US and Europe than in China (Fig 4B). The prevalence of death was 23% [95% CI: 19–27%; I^2^ = 97%; n = 29] in the US and Europe, and 11% [95% CI: 7–16%; I^2^ = 94%; n = 24] in China. Prevalence of severe disease was 20% [95% CI: 16–25%; I^2^ = 98%; n = 25] for US and Europe, and 39% [95% CI: 32–47%; I^2^ = 97%; n = 30] for China. Median age of patients was 65 years [IQR: 63–67 years; I^2^ = 0%; n = 24] for the US and Europe and 55 years [IQR: 52–58 years; I^2^ = 57%; n = 27] for China. Fifty-two percent [95% CI: 46–59%; I^2^ = 98%; n = 16] of the patients hospitalized were aged ≥60 years in the US and Europe as compared to 36% [95% CI: 30–43%; I^2^ = 96%; n = 22] for China. The prevalence of co-morbidities between US-Europe and China differed as follows: **1) US-Europe**: HTN = 55% [95% CI: 52–57%]; diabetes = 31% [95% CI: 29–34]; CVD = 18% [95% CI: 15–21%]; smoking history = 15% [95% CI: 11–21%]; COPD = 9% [95% CI: 6–13%] and **2) China**: HTN = 23% [95% CI: 20–26%]; diabetes = 12% [95% CI: 10–14%]; CVD = 16% [95% CI: 12–22%]; smoking history = 11% [95% CI: 9–13%]; CKD = 2.3% [95 CI: 1.6–3.4%] and COPD = 4% [95 CI: 3–5%].

### Comorbidities in COVID-19 patients and the general populations in the US and China

In order to gain some understanding of whether patients with comorbidities are at higher risk of COVID-19 infection or hospitalization, we compared the prevalence of comorbidities between COVID-19 patients hospitalized in the US and the prevalence of comorbidities in the general US population. We observed that the prevalence of hypertension (55%), diabetes (33%), CVD (17%), and smoking history (23%) were substantially higher in the COVID-19 patients than in the general US population (Fig 4C). For the Chinese population, the overall prevalence of hypertension (23%) and diabetes (12%) in the COVID-19 patients were similar to that of the general Chinese population. However, the prevalence of smoking history (11%), COPD (4%), CKD (2%), and heart disease (16%) in the COVID-19 patients hospitalized in China were unexpectedly lower as compared to their corresponding prevalence in the general Chinese population (Fig 4D).

### Sensitivity analyses

The positive associations of age≥65 years, male sex, smoking history, COPD, hypertension and diabetes with the risk of death in the COVID-19 patients were relatively homogenous (I^2^<70%). However, we carried out sensitivity analyses to assess the effects of outliers. For the risk of death for hypertension and smoking history, we removed the study by Yao et al. [86] which showed significantly higher risk compared to other studies; the results for both hypertension [sRR = 1.74; 95% CI: 1.58–1.94] and smoking [sRR:1.24; 95% CI: 1.08–1.42] remained significant. Guan et al. [13] had published a second study with additional patients and reported adjusted estimates for COPD, diabetes and hypertension. We used the adjusted risk estimates for the analyses. For the risk of death with other risk factors (CVD, CKD, and CLD) for Guan et al. [45], we conducted sensitivity analyses by using the counts only from the original study. The results [sRR (95% CI)] were similar as: CVD = 2.06 [95 CI: 1.80–2.36], CKD = 2.48 [95% CI: 2.09–2.94] and CLD = 2.67 [95% CI: 1.85–3.85].

### Small study effects and quality assessment

We observed asymmetry in the funnel plot for studies that reported prevalence of death in COVID-19 patients (Egger’s test p = 0.001) (S1 Fig). On further analysis, the plot remained asymmetrical when restricted to studies from China (Egger’s p = 0.003) but was symmetrical for studies from US-Europe (Egger’s p = 0.160). We observed symmetrical funnel plots with no bias for pooled prevalence severe disease (Egger’s p = 0.128). On average, prospective or retrospective studies scored a score of 6 out of 9 and cross-sectional studies scored 6 out of 10. Many studies did not get a full score because they did not adjust for confounders (age, sex, or other risk factors) or patients remained hospitalized even after the follow-up ended, suggesting inadequate follow-up period (S4 Table).

## Discussion

We carried out a comprehensive systematic review and meta-analysis of 77 studies that included 38906 hospitalized patients to investigate the prevalence and risk factors for death and severe disease in COVID-19 patients. We calculated an overall prevalence of death of 20% and severe disease of 28%. Nearly 50% of the patients admitted to hospitals due to COVID-19 were ≥60 years of age and 59% were males. We observed high prevalence of hypertension and diabetes of 50% and 28%, respectively, for the patients. The risk factors were more prevalent in patients who died and were distributed as: age ≥60 years: 85%; males: 66%; hypertension: 66%; diabetes: 39%; heart disease: 37%; CKD: 27%; smoking history: 44%; COPD: 12%, and CLD: 9%. In comparison with the overall prevalence of death of 20% for all COVID-19 hospitalized patients, the CFR was higher for male patients (26%) and for patients having the following risk factors: age≥60 years (35%), heart disease (52%), COPD (51%), CKD (48%), CLD (39%), hypertension (28%), diabetes (24%), and smoking history (27%). The elevation in the risk of death was statistically significant for age ≥60 (sRR = 3.6; 95% CI: 3.0–4.4), male sex 1.3 (95% CI: 1.2–1.4), smoking history (sRR = 1.3; 95% CI: 1.1–1.6), COPD (sRR = 1.7; 95% CI: 1.4–2.0), heart disease (sRR = 2.1; 95% CI: 1.8–2.4), CKD (sRR = 2.5; 95% CI: 2.1–3.0), hypertension (sRR = 1.8; 95% CI: 1.7–2.1), and diabetes (sRR = 1.5; 95% CI: 1.4–1.7). All of the risk factors we analyzed were positively associated with progression to severe disease as well. The results suggest that older age, male sex and the co-morbidities increase the risk of progression to severe disease and death in COVID-19 patients.

We observed significant difference in the prevalence of death between US-Europe (23%) and China (11%). This lower risk of death from COVID-19 for the hospitalized patients in China may be explained by the lower median age as well as lower prevalence of co-morbidities for COVID-19 patients in China. However, this >200% lower prevalence of death in China is incommensurate with our finding of a higher prevalence of severe disease observed for patients in China (39%) as compared to patients in the US-Europe (20%). Notably, we observed asymmetry in the funnel plot and a statistically significant tests for publication bias or small study effects for the prevalence of death for studies from China that could suggest selective outcome reporting. As such, while the lower median age and prevalence of co-morbidities for COVID-19 patients in China may explain the lower prevalence of death, it is also possible that a selective under-reporting of death had occurred for studies from China. The death toll in China was initially under-reported and later updated on April 17, 2020 [95].

Whether or not cigarette smoking has been associated with SARS-CoV-2 acquisition or progression to severe disease has been strongly debated with studies showing both positive, null, and inverse association between smoking and COVID-19 [10, 11, 96–98]. We found that patients with any history of smoking have both a higher risk of death (RR: 1.28; 95% CI: 1.06–1.55) and severe disease (1.29; 95% CI: 1.18–1.42). The case fatality risk for those with smoking history (27%) was also higher than the overall CFR of 20%. Whereas a higher COVID-19 mortality and morbidity among smokers may be due its causal association with COPD and CVD, Cai et al. [99] has also observed upregulation of pulmonary Angiotensin Converting Enzyme 2 (ACE2) gene expression and hence, pulmonary ACE2 receptors in smokers suggesting a direct effect of smoking on COVID-19 susceptibility and disease progression. ACE2 receptors are used by SARS-CoV-2 to translocate intracellularly [15, 100–104].

Our results of higher risk of death and severe disease associated with hypertension, diabetes and CVD in COVID-19 patients concurred with most studies conducted to date including studies that specifically investigated these associations [14, 65, 105, 106]. However, it is unclear if cardiovascular risk factors including smoking, hypertension, diabetes, heart disease and CKD increases the susceptibility toward SARS-CoV-2 infection in the population [15, 100, 101, 107]. On one hand, angiotensin-converting enzyme 2 (ACE2)–by blocking the renin angiotensin aldosterone system (RAAS) and decreasing or countering the vasoconstrictive, proinflammatory and profibrotic properties of angiotensin-II through catalysis of angiotensin-II to angiotensin-(1–7)–have been shown to exert cardiovascular protective effect and prevent acute lung injury from SARS-CoV-2 [15, 100, 101]. However, on the other hand, a possible greater expression of ACE2, the functional receptor mediating cellular entry of SARS-CoV-2 in humans, in patients with cardiovascular disease and other comorbidities can lead to increased susceptibility towards infection with SARS-CoV-2 [108, 109]. In this context, it would be reasonable to posit that a substantially higher prevalence of cardiovascular comorbidities in the hospitalized patients compared to the prevalence in the general population may suggest elevated risk of acquisition of SARS-CoV-2 for patients with cardiovascular risk factors. To this end, we found that the prevalence of smoking history (23%), hypertension (55%), diabetes (33%) and heart disease (17%) in the hospitalized COVID-19 patients in the US were substantially higher than the corresponding prevalence of smoking (14%) [110], hypertension (29%) [111], diabetes (13%) [112] and heart disease (9%) [113] in the general US population that could suggest an association between these comorbidities and risk of SARS-CoV-2 infection or disease progression. However, we note that if the prevalence of these comorbidities in the asymptomatic individuals with COVID-19 in the general population is similar to that of their prevalence in the non-COVID-19 general population, then this difference–the higher prevalence of comorbidities in the hospitalized patients compared to the general population–could simply imply a higher risk of symptomatic infection or hospitalization for individuals having SARS-CoV-2 infection. The prevalence of other risk factors i.e. COPD (9%) and CKD (15%) in the COVID-19 patients in the US was similar to the overall prevalence of COPD (7%) [114] and CKD (15%) [115] in the country. Generally, we noted a lower prevalence of comorbidities for patients in China. The prevalence of hypertension (23%) and diabetes (12%) in the hospitalized patients in China, which were lower than that of the US, approximate the respective prevalence of hypertension (23%) [116] and diabetes (15%) [117] in the general population of China. A previous meta-analysis also noted this observation [19]. Surprisingly, the prevalence of smoking (11%) in the COVID-19 patients hospitalized in China are inexplicably lower than the corresponding prevalence of smoking (23%) among COVID-19 patients in the US despite a higher prevalence of smoking (47% in Chinese males) [118] in the general Chinese population is significantly higher than that of the US. The prevalence of CVD (16%), COPD (4%) and CKD (2%) among COVID-19 patients in China are substantially lower than the corresponding prevalence of CVD (21%) [119], COPD (14%) [120], and CKD (11%) [121] in the general Chinese population. Given these discrepancies, we are unsure whether the lower prevalence of comorbidities noted for the COVID-19 patients in China are representative of the true prevalence. There was a great sense of urgency and a race to publish data in the early phase of the outbreak. As such, there exists the possibility of substantial under-recording of data on covariables. Had there been under-reporting, the implication would be a higher true prevalence estimate. We do not see reason for any systematic difference in reporting of risk factors based on outcome, or vice-versa, and hence, our summary relative risk estimates for association of risk factors with death or severe disease should not have been affected.

We assessed if patients with specific co-morbidities at baseline had higher risk of specific organ injury from SARS-CoV-2 during hospitalization. While the available data did not allow direct assessment of this relation, we compared the prevalence of comorbidities with the prevalence of corresponding organ system injury for studies that reported both baseline comorbidity and corresponding organ injury. We observed that the risk of acute lung injury/ARDS (48%), ACI (21%), and AKI (27%) were substantially higher than the baseline prevalence of COPD (6%), heart disease (11%) and CKD (14%), respectively. The higher prevalence of acute organ injury than the prevalence of baseline comorbidity simply indicates that ARDS, ACI and AKI were also occurring in patients who did not have a corresponding comorbidity at baseline in addition to people having the comorbidities.

Most studies reported only frequencies of risk factors and did not present adjusted measures for disease severity or death. Given this limitation, the risk ratio we calculated from the frequencies are largely unadjusted estimates. Future studies could additionally present, at the least, age- and sex-adjusted measures for association of risk of comorbidities with death or severe disease. Many studies reported odds ratio for the measure of association between pre-existing conditions and risk of severe disease or death. Odds ratio poorly approximates risk ratio when the disease prevalence is high at baseline. For example, Zhou et al. [14] calculated an odds ratio of 5.4 (95% CI: 0.96–30.4) for risk of death from COPD in COVID-19 patients whereas the risk ratio we calculated from the frequencies presented is RR = 2.47 (95% CI: 1.34–4.55). Prevalence of severe disease or death in COVID-19 patients was high in several studies. Similarly, several meta-analyses calculated odds ratios instead of risk ratios to summarize the risk of disease severity or death in association with risk factors such as smoking, diabetes, hypertension and cardiovascular disease [10, 11, 18], often to be interpreted by media and even by researchers as a measure of relative risk. Lack of rigor in research design, analysis and interpretation could generate inconsistent and ungeneralizable results across studies leading to controversy and confusion around serious public health issues such as that existing for association (or not) of smoking with COVID-19 disease acquisition, severity or death. As publications evolve at a pace that could be overwhelming for researchers and practitioners, we attempted to present a meaningful summary and inference for association of risk factors with death or severe disease from literatures published globally. Additionally, we provide an epidemiological framework for the risk of infection by SARS-CoV-2 based on presence of cardiovascular risk factors. This analysis can inform public health measures for COVID-19 screening and prevention, risk stratification and management of patients in clinical practice, analysis and presentation strategies for research data and inspire etiological investigations.

## Conclusion

Epidemiological risk factors for progression of COVID-19 to severe disease and death and for acquisition of SARS-CoV-2, the causal agent for COVID-19, based on presence of pre-existing conditions have been insufficiently understood. Meta-analysis of 77 studies including 39023 COVID-19 patients hospitalized globally revealed case fatality risk of 52% for those having heart disease, 51% for COPD, 48% for CKD, 39% for CLD, 28% for hypertension, 27% for smoking history, 24% for diabetes, 35% for age≥60 years, and 26% for males. Of all the patients who died, an overwhelming majority (85%) were in people aged≥60 years. Also, of the people who died, 66% were males, 66% had hypertension, 44% had history of smoking, 39% had diabetes, 37% had CVD, 27% had CKD, and 6% had CLD. All of the above risk factors were significantly associated with death and severe disease in the patients hospitalized for COVID-19. The prevalence of ARDS was 48%, ACI 21%, and AKI 28% in the hospitalized patients. A higher prevalence of hypertension, diabetes, smoking and heart disease in the COVID-19 inpatients as compared to that of the general population could imply a higher risk of SARS-CoV-2 infection or disease progression for patients having these risk factors. These findings could inform public health strategies for targeted screening and appropriate control of modifiable risk factors such as smoking, hypertension, and diabetes to reduce morbidity and mortality. Finally, based on the published literature, there were vast differences in the prevalence of death and risk factors for the populations in China and in US-Europe that should be further investigated.

## Supporting information

S1 Table Prevalence of death, severe disease and risk factors in COVID-19 patients (December 2019-August 2020). (DOCX)

S2 Table Prevalence of death stratified by risk factors in COVID-19 patients (December 2019-August 2020). (DOCX)

S3 Table Prevalence of severe disease stratified by risk factors in COVID-19 patients (Dec 2019-August 2020). (DOCX)

S4 Table Newcastle-Ottawa quality assessment (modified) for studies^#^. ^#^Award of Points: Selection: points were awarded based on representativeness of the exposed group and unexposed group (2 points), ascertainment of exposures (1 point), and demonstration that outcome of interest was not present at the start of the study (1 point). Comparability (2 points): points were awarded based on whether the analyses were adjusted for age, sex, and other risk factors (2 points for adjustment to age and sex). Outcome (3points): points were awarded based on ascertainment of outcome through record linkage or independent blind assessment (1 points); duration of follow-up (1 point) (hospitalization till discharge); and adequacy of follow up for study population (complete follow up for the patients (vs whether patients were currently under treatment at the time of study report) (1 point), or if the patients currently under admission are excluded from outcome assessment (1 point).
(DOCX)

S1 Fig Publication bias or small study effects for prevalence of death and severe disease. (TIF)

S1 Checklist PRISMA 2009 checklist. (DOC)
