# Supplementary material for: Prevalence and predictors of death and severe disease in patients hospitalized due to COVID-19: A comprehensive systematic review and meta-analysis of 77 studies and 38,000 patients
Source: PLoS One. 2020 Dec 7;15(12):e0243191. doi: 10.1371/journal.pone.0243191 (PMC7721151; doi:10.1371/journal.pone.0243191)
Supplement: S1 Table — (DOCX) [file pone.0243191.s001.docx]

S1 Table. Prevalence of death, severe disease and their risk factors in persons hospitalized with SARS Cov-2 (December2019-May 2020)

| **Author, year** | **Median age, years (IQR)** | **Male sex,**  **% (n/N)** | **Age≥65 years**  **% (n/N)** | **Smoking,**  **% (n/N)** | **Hypertension% (n/N)** | **Diabetes,**  **% (n/N)** | **CVD,**  **% (n/N)** | **COPD,**  **% (n/N)** | **CKD,**  **% (n/N)** | **CLD,**  **% (n/N)** | **Death, % (n/N)** | **Severe disease, % (n/N)** |
| --- | --- | --- | --- | --- | --- | --- | --- | --- | --- | --- | --- | --- |
| Aggarwal S et al., 2020 | 67  (38-95) | 75  (12/16) |  | 0  (0/16) | 56  (9/16) | 31  (5/16) | 44  (7/16) | 13  (2/16) | 38  (6/16) |  | 19  (3/16) | 50  (8/16) |
| Cao J et al.,  2020 | 54  (37-67) | 52  (53/102) |  |  | 27  (28/102) | 11  (11/102) | 5  (5/102) |  | 4  (4/102) | 2  (2/102) | 17  (17/102) | 18  (18/102) |
| CDC (USA), 2020 |  |  | 51  (715/1389) | 7  105/1494) |  | 26  (399/1494) | 25  (374/1494) | 16  (246/1494) | 10  (151/1494) | 1  (16/1494) |  | 13  (612/4860) |
| Chen G et al., 2020 | 56  (50-65) | 81  (17/21) |  |  | 24  (5/21) | 14  (3/21) |  |  |  |  | 0  (0/21) | 52  (11/21) |
| Chen J et al., 2020 | 51  (36-64) | 51  (126/249) |  |  |  |  | 22  (55/249) |  |  | 1  (2/249) | 1  (2/249) | 9  (22/249) |
| Chen Q et al., 2020 | 48  (38-55) | 54  (79/145) |  | 10  (15/145) | 14  (21/145) | 10  (14/145) | 1  (1/145) | 4  (6/145) | 2  (3/145) | 3  (4/145) |  | 30  43/145) |
| Chen T et al., 2020 | 62  (44-70) | 62  (171/274) |  | 7  (19/274) | 34  (93/274) | 17  (47/274) | 8  (23/274) | 7  (18/274) | 1  (4/274) | 4  (11/274) |  |  |
| Cheng Y et al., 2020 | 63  (50-71) | 52  (367/704) |  |  |  |  |  |  | 14  (101/698) |  | 16  (113/701) | 10  (73/701) |
| Deng Y et al., 2020 | 69  (62-74) | 55  (124/225) |  |  | 26  (58/225) | 12  (28/225) | 8  (17/225) |  |  |  |  |  |
| Du R-H et al., 2020 | 58  (49-67) | 54  (97/179) | 36  (65/179) |  | 32  (58/179) | 18  (33/179) | 16  (29/179) |  |  |  | 12  (21/179) |  |
| Feng Y et al., 2020 | 54  (40-64) | 57  (271/476) | 25  (118/476) | 10  (44/454) | 24  (113/476) | 10  (49/476) | 8  (38/476) | 5  (22/476) | 1  (4/476) |  | 8  (38/476) | 26  (124/476) |
| Ferguson J et al., 2020 | 60  (43-71) | 53  (38/72) |  | 24  (17/72) | 36  (26/72) | 28  (20/72) | 10  (7/72) | 14  (10/72) | 13  (9/72) |  | 7  (5/72) | 29  (21/72) |
| Gold J et al., 2020 | 60  (46-69) | 50  (151/305) | 38  (117/305) |  | 68  (206/305) | 40  (121/305) | 26  (78/305) | 5  (16/305) | 16  (48/305) | 0  (7/305) | 16  (48/305) | 28  (86/305) |
| Goyal S et al., 2020 | 62  (49-74) | 61  (238/393) |  | 5  (20/393) | 50  (197/393) | 25  (99/393) | 14  (54/393) | 5  (20/393) |  |  | 10  (40/393) | 33  (130/393) |
| Guan W et al., 2020 | 47 (35-58) | 61  (637/1096) | 14  (153/1096) | 15  (158/1085) | 15  (165/1099) | 7  (81/1099) | 2  (27/1099) | 1  (12/1099) |  | 2  (23/1099) | 6  (67/1099) | 16  (173/1099) |
| Guan Wei-Jie, 2020 (ERJ) | 49 (38-60) | 59  (904/1590) |  | 7  (111/1590) | 17  (269/1590) | 8  (130/1590) | 4  (59/1590) | 2  (24/1590) | 1  (21/1590) | 2  (28/1590) | 3  50/1590) | 8  (131/1590) |
| Hu L et al., 2020 | 61  (23-91) |  | 34  (111/323) | 12  (38/323) | 33  105/323) | 15  (47/323) | 13  (41/323) | 2  (6/323) | 2  (7/323) | 2  (5/323) |  | 53  (172/323) |
| Huang C et al., 2020 | 49 (41-58) | 73  (30/41) |  | 7  (3/41) | 15  (6/41) | 20  (8/41) | 15  (6/41) | 2  (1/41) |  | 2  (1/41) | 15  (6/41) | 32  (13/41) |
| Inciardi R et al., 2020 | 67  (59-75) | 81  (80/99) |  | 17  (17/99) | 64  (63/99) | 30  (30/99) | 16  (16/99) | 9  (9/99) | 15  (15/99) |  | 26  (26/99) | 37  (37/99) |
| Javanian M et al., 2020 | 60  (51-69) | 51  (51/100) |  |  | 32  (32/100) | 37  (37/100) | 20  (20/100) | 12  (12/100) | 12  (12/100) | 3  (3/100) | 19  (19/100) | 4  (4/100) |
| Li X et al., 2020 | 60  (48-69) | 51  (279/548) | 38  (210/548) | 17  (92/544) | 30  (166/548) | 15  (83/548) | 6  (34/548) | 3  (17/548) | 2  (10/548) |  | 16  (87/548) | 49  (269/548) |
| Liu W et al., 2020 | 38 (33-57) | 50  (39/78) | 19  15/78 | 6  (5/78) | 10  (8/78) | 6  (5/78) |  | 3  (2/78) |  |  | 3  (2/78) | 14  (11/78) |
| Nowak B et al., 2020 | 64  (51-77) | 51  (87/169) |  |  | 47  (80/169) | 19  (32/169) | 34  (58/169) | 13  (22/169) | 21  (35/169) |  | 27  (46/169) | 16  (27/169) |
| Palaiodimos L et al., 2020 | 64  (50-74) | 49  (98/200) | 48  (96/200) | 33  (65/200) | 76  (152/200) | 40  (79/200) | 17  (33/200) | 14  (28/200) | 21  (41/200) | 1  (2/200) | 24  (48/200) | 39  (77/200) |
| Richardson S et al., 2020 | 63  (52-75) | 60  (3437/5700) | 45  (2582/5700) | 16  (558/3567) | 53  (3026/5700) | 32  (1808/5700) | 10  (595/5700) | 5  (287/5700) | 5  (268/5700) | 0  (11/5700) | 21  (553/2634) | 14  (373/2634) |
| Shi Y et al., 2020 | 46  (33-59) | 53  (259/487) |  | 8  (40/487) | 20  (99/487) | 6  (29/487) | 2  (11/487) |  | 1  (7/487) | 5  (22/487) |  | 10  (49/487) |
| Tian S et al., 2020 |  | 49  (127/262) | 18  48/262 |  |  |  |  |  |  |  | 1  (3/262) | 18  (46/262) |
| Tomlins J et al., 2020 | 75  (59-82) | 63  (60/95) |  |  | 37  (35/95) | 39  (37/95) | 20  (19/95) | 11  (10/95) | 23  (22/95) |  | 21  (20/95) |  |
| Wan S et al., 2020 | 47 (36-55) | 16  (21/135) |  | 7  (9/135) | 10  13/135 | 9  (12/135) | 5  (7/135) |  |  |  | 1  (1/135) | 30  (40/135) |
| Wang D et al., 2020 | 56 (42-68) | 54  (75/138) |  |  | 31  (43/138) | 10  (14/138) | 14  (20/138) | 3  (4/138) | 3  (4/138) | 3  (4/138) | 4  (6/138) | 26  (36/138) |
| Wang R et al., 2020 | 39  (30-48) | 57  (71/125) |  | 13  (16/125) |  |  | 14  (18/125) |  |  |  |  | 20  (25/125) |
| Wang Z et al., 2020 | 42 (35-62) | 46  (32/69) |  |  | 13  (9/69) | 10  (7/69) | 12  (8/69) | 6  (4/69) |  | 1  (1/69) | 7  (5/69) | 20  (14/69) |
| Wu C et al., 2020 | 51 (43-60) | 57  (128/201) | 20  (40/201) |  | 19  (39/201) | 11  (22/201) | 4  (8/201) |  | 1  (2/201) | 3  (7/201) | 22  (44/201) | 42  (84/201) |
| Yao Q et al., 2020 | 52  (37-58) | 40  (43/108) | 16  (17/108) | 4  (4/108) | 15  (16/108) | 5  (5/108) | 4  (4/108) |  |  | 2  (2/108) | 11  (12/108) | 12  (13/108) |
| Young BE et al., 2020 | 47 (31-73) | 50  (9/18) |  |  |  |  |  |  |  |  |  | 50  (9/18) |
| Yu T et al., 2020 |  | 15  (14/95) |  |  |  |  |  |  |  |  |  | 25  (24/95) |
| Yu X et al., 2020 |  | 52  (172/333) | 32  (107/333) | 8  (26/333) | 19  (64/333) | 8  28/333) | 7  (24/333) |  |  |  |  | 8  (26/333) |
| Zhang G et al., 2020 | 49 (39-58) | 56  (53/95) |  |  |  |  |  |  |  |  | 6  (6/95) | 34  (32/95) |
| Zhang JJ et at., 2020 | 57 (25-87) | 51  (71/140) | 21  (29/140) | 6  (9/140) | 30  (42/140) |  |  |  | 1  (2/140) | 6  (8/140) |  | 41  (58/140) |
| Zhao X-Y et al., 2020 |  | 54  (49/91) | 18  (16/91) |  |  | 3  (3/91) |  | 1  (1/91) | 1  (1/(1) |  | 2  (2/91) | 33  (30/91) |
| Zheng S et al., 2020 | 55 (44-65) | 60  (58/96) |  |  | 36  (35/96) | 11  (11/96) | 7  (7/96) | 4  (4/96) | 1  (1/96) | 3  (3/96) | 0  (0/96) | 31  (30/96) |
| Zheng Y et al., 2020 |  | 55  (40/73) | 14  (10/73) | 11  (8/73) | 12  (9/73) | 5  (4/73) | 3  (2/73) |  |  | 3  (2/73) |  | 41  (30/73) |
| Zhou F et al., 2020 | 56 (46-67) | 62  (119/191) |  | 6  (11/191) | 30  (58/191) | 19  (36/191) | 8  (15/191) | 3  (6/191) | 1  (2/191) |  | 28  (54/191) | 26  (50/191) |
| Turcotte J et al., 2020 | 66 (55-77) | 53 (62/117) |  | 37 (43/117) | 66 (77/117) | 39 (46/117) | 22 (26/117) | 29 (34/117) | 23 (27/117) |  | 25 (29/117) | 31 (36/117) |
| Zhang J et al., 2020 | 56 (44-69) | 48 (321/663) | 48 (315/663) |  |  |  | 25 (164/663) |  |  |  | 4 (25/663) | 62 (409/663) |
| Nikpouraghdam M. 2020 | 56 (46-65) | 66 (1955/2964) | 39 (1164/2964) |  | 2 (59/2964) | 4 (113/2964) | 1 (37/2964) | 2 (60/2964) |  |  | 8 (239/2964) |  |
| Khalil K 2020 |  | 59 (130/220) | 53 (117/220) | 40 (88/220) | 45 (99/220) | 28 (61/220) | 11 (24/220) | 9 (20/220) | 7 (16/220) | 3 (6/220) | 26 (58/220) |  |
| Iaccarino G 2020 |  | 64 (1018.24/1591) |  |  | 55 (873.459/1591) | 17 (268.879/1591) | 14 (216.376/1591) | 8 (122.507/1591) | 6 (87.505/1591) |  | 12 (188/1591) | 15 (235/1591) |
| Kalligeros M 2020 | 60 (50-72) | 61 (63/103) |  | 47 (48/103) | 64 (66/103) | 37 (38/103) | 24 (25/103) | 19 (20/103) | 11 (11/103) | 3 (3/103) |  | 43 (44/103) |
| Cao Z 2020 | 53 (40-66) | 48 (38/80) |  | 6 (5/80) | 25 (20/80) | 8 (6/80) | 13 (10/80) | 6 (5/80) |  |  | 4 (3/80) | 34 (27/80) |
| Hsu H 2020 |  | 48 (1312/2729) | 34 (928/2729) |  | 46 (1248/2729) | 26 (708/2729) | 7 (190/2729) | 5 (146/2729) | 16 (438/2729) |  | 4 (98/2729) | 7 (188/2729) |
| Hur K 2020 |  | 56 (271/486) | 45 (217/486) | 34 (163/486) | 55 (267/486) | 33 (160/486) | 23 (111/486) | 16 (78/486) | 9 (42/486) |  | 4 (21/486) | 28 (138/486) |
| Shabrawishi M 2020 | 56 (36-56) | 60 (90/150) |  |  | 28 (42/150) | 25 (38/150) | 7 (11/150) | 1 (1/150) | 7 (10/150) | 1 (1/150) | 3 (4/150) | 11 (16/150) |
| Giacomelli A 2020 | 61 (50-72) | 31 (72/233) | 42 (99/233) | 30 (70/233) |  |  |  |  |  |  | 21 (48/233) | 3 (8/233) |
| Garibaldi B 2020 | 63 (49-75) | 53 (443/832) |  | 29 (239/832) | 47 (389/832) | 30 (252/832) | 32 (266/832) | 18 (151/832) | 13 (106/832) | 4 (34/832) | 16 (131/832) | 21 (171/832) |
| Zhan et al., 2020 |  | 55 (221/405) | 46 (186/405) | 11 (46/405) |  | 22 (88/405) | 39 (156/405) | 8 (31/405) | 7 (28/405) |  |  | 37 (148/405) |
| Wei et al., 2020 | 51 (41-58) | 56 (155/276) | 22 (60/276) | 13 (28/220) | 17 (47/276) | 5 (14/276) | 4 (12/276) | 3 (7/276) |  |  |  | 5 (14/276) |
| Sun L et al., 2020 | 44 (34-56) | 56 (31/55) |  |  |  |  | 2 (1/55) |  |  |  |  | 27 (15/55) |
| Khamis F et al., 2020 | 48 (35-61) | 84 (53/63) |  | 6 (4/63) | 32 (20/63) | 32 (20/63) | 6 (4/63) | 6 (4/63) | 6 (4/63) |  | 8 (5/63) | 38 (24/63) |
| Hewitt J et al., 2020 | 74 (61-83) | 58 (903/1564) | 69 (1076/1564) | 46 (724/1564) | 51 (804/1564) | 27 (415/1564) | 22 (345/1564) |  |  |  | 27 (425/1564) |  |
| Escalera-Antezana J et al., 2020 | 44 (33-55) | 53 (57/107) | 29 (31/107) |  | 12 (13/107) | 7 (7/107) | 3 (3/107) |  |  |  | 6 (6/107) |  |
| Gregoriano et al., 2020 | 67 (56-76) | 63 (62/99) |  | 8 (6/76) | 57 (56/99) | 22 (22/99) | 28 (28/99) | 7 (7/99) | 28 (28/99) |  | 18 (18/99) | 35 (35/99) |
| Chilimuri S et al., 2020 | 63 (52-72) | 63 (236/375) |  |  | 60 (225/375) | 47 (175/375) | 17 (62/375) | 17 (62/375) | 14 (51/375) | 5 (18/375) | 43 (160/375) |  |
| Pellaud et al., 2020 | 70 (60-80) | 61 (119/196) | 76 (148/196) | 29 (56/196) | 60 (118/196) | 27 (52/196) | 13 (26/196) | 8 (16/196) |  |  | 17 (33/196) | 25 (49/196) |
| Brill S et al., 2020 | 72 (56-83) | 60 (272/450) | 70 (313/450) |  | 43 (195/450) | 30 (134/450) | 31 (141/450) |  |  |  | 38 (173/450) |  |
| Rivera-Izquierdo et al., 2020 | 67 (57-77) | 55 (131/238) | 61 (144/238) | 26 (62/238) | 49 (116/238) | 22 (52/238) | 23 (54/238) |  | 10 (23/238) |  | 26 (61/238) |  |
| Shahriarirad R et al., 2020 | 54 (43-67) | 63 (71/113) | 25 (28/113) |  | 19 (22/113) | 14 (16/113) | 14 (16/113) | 8 (9/113) | 5 (6/113) |  | 8 (9/113) | 10 (11/113) |
| Liu S et al., 2020 |  | 53 (329/625) | 13 (79/625) |  | 15 (91/625) | 6 (40/625) |  |  |  |  | 0 (0/625 | 10 (64/625) |
| Tambe et al., 2020 | 46 (34-57) |  |  |  | 30 (60/197) | 21 (42/197) | 2 (4/197) | 5 (10/197) | 1 (2/197) |  | 29 (58/197) |  |
| Suleyman et al., 2020 | 61 (51-72) | 46 (165/355) | 57 (202/355) | 39 (137/355) | 73 (258/355) | 44 (156/355) | 16 (56/355) | 12 (41/355) | 45 (161/355) |  | 20 (72/355) | 40 (141/355) |
| Shekhar et al., 2020 |  | 46 (23/50) | 28 (14/50) | 48 (24/50) | 34 (17/50) | 36 (18/50) |  |  |  | 4 (2/50) | 26 (13/50) | 68 (34/50) |
| Okoh et al., 2020 | 62 (49-74) | 51 (129/251) |  |  | 70 (175/251) | 46 (115/251) | 20 (49/251) | 9 (23/251) | 18 (46/251) |  | 39 (97/251) | 33 (82/251) |
| Lendorf et al., 2020 | 68 (56-78) | 60 (67/111) |  | 39 (43/111) | 34 (38/111) | 14 (16/111) | 17 (19/111) | 7 (8/111) | 7 (8/111) |  | 14 (16/111) | 18 (20/111) |
| Jang et al., 2020 | 57 (45-68) | 44 (48/110) |  |  | 34 (37/110) | 26 (29/110) | 9 (10/110) | 4 (4/110) |  | 1 (1/110) | 7 (8/110) | 21 (23/110) |
| Galloway et al., 2020 | 71 (57-82) | 58 (666/1157) |  |  | 53 (611/1156) | 35 (408/1156) | 13 (152/1155) | 20 (234/1156) | 16 (189/1156) |  | 21 (244/1157) | 14 (157/1157) |
| Ciceri et al., 2020 | 65 (56-75) | 73 (299/410) | 50 (207/410) |  | 50 (203/410) | 17 (69/410) | 12 (51/410) | 5 (22/410) | 11 (47/410) |  | 23 (95/410) |  |
| Argenziano et al., 2020 | 63 (50-75) | 60 (511/850) | 46 (388/850) | 23 (198/850) | 62 (525/850) | 39 (333/850) | 14 (115/850) | 7 (56/850) | 15 (125/850) | 2 (17/850) | 25 (211/850) | 28 (236/850) |

Note: cells are shaded for no data in the study.
