# Supplementary material for: Prevalence and predictors of death and severe disease in patients hospitalized due to COVID-19: A comprehensive systematic review and meta-analysis of 77 studies and 38,000 patients
Source: PLoS One. 2020 Dec 7;15(12):e0243191. doi: 10.1371/journal.pone.0243191 (PMC7721151; doi:10.1371/journal.pone.0243191)
Supplement: S2 Table — (DOCX) [file pone.0243191.s002.docx]

S2 Table. Prevalence of death in risk groups and prevalence of risk factor in COVID-19 patients who died during hospitalization (Dec 2019-May 2020)

| Author, year | Male sex, % (n/N) | | Age≥60  % (n/N) | | Smoking history (SH),  % (n/N) | | Hypertension (HTN), % (n/N) | | Diabetes mellitus (DM),  % (n/N) | | Cardiovascular disease (CVD),  % (n/N) | | Chronic Obstructive Pulmonary Disease (COPD),  % (n/N) | | Chronic Kidney Disease (CKD),  % (n/N) | | Chronic liver disease,  % (n/N) | |
| --- | --- | --- | --- | --- | --- | --- | --- | --- | --- | --- | --- | --- | --- | --- | --- | --- | --- | --- |
|  | Death in male/Total male | Death in male/Total death | Death in >60/  Total>60 | Death in >60/  Total death | Death in SH/  Total SH | Death in SH/Total death | Death in HTN/Total HTN | Death in HTN/Total death | Death in DM/Total DM | Death in DM/Total death | Death in CVD/Total CVD | Death in CVD/Total CVD | Death in COPD/Total COPD | Death in COPD /Total COPD | Death in CKD/Total CKD | Death in CKD/Total CKD | Death in CLD/Total CLD | Death in CLD/Total CLD |
| Chen T et al., 2020 | 49  (83/171) | 73  (83/113) | 61  (94/153) | 83  (94/113) |  |  | 58  (54/93) | 48  (54/113) | 51  (24/47) | 21  (24/113) | 70  (16/23) | 14  (16/113) | 61  (11/18) | 10  (11/113) | 80  (4/5) | 4  (4/113) | 45  (5/11) | 4  (5/113) |
| Deng Y et al., 2020 | 59  (73/124) | 67  (73/109) |  |  |  |  | 69  (40/58) | 37  (40/109) | 61  (17/28) | 16  (17/109) | 76  (13/17) | 12  (13/109) |  |  |  |  |  |  |
| Du R-H et al., 2020 | 10  (10/97) | 48  (10/21) | 26  (17/65) | 81  (17/21) |  |  | 22  (13/58) | 62  (13/21) | 18  (6/33) | 29  (6/21) | 41  (12/29) | 57  (12/21) |  |  |  |  |  |  |
| Gold J et al., 2020 |  |  | 31  (36/117) | 75  (36/48) |  |  |  |  |  |  |  |  |  |  |  |  |  |  |
| Guan W et al., 2020 | 7  (45/637) | 67  (45/67) | 21  (32/153) | 49  (32/65) | 14  (22/158) | 33 (22/66) | 15  (24/165) | 37 (24/67) | 22  (18/81) | 27 (18/67) | 22  (6/27) | 9  (6/67) | 58  (7/12) | 10 (7/67) | 25  (2/8) | 3  (2/67) | 4  (1/23) | 1  (1/67) |
| Guan Wei-Jie, 2020 (ERJ) |  |  |  |  |  |  | 10  (28/269) | 56 (28/50) | 10  (13/130) | 26 (13/50) | 14  (8/59) | 16  (8/50) | 25  (6/24) | 12 (6/50) | 24  (5/21) | 10  (5/50) | 4  (1/28) | 2  (1/50) |
| Inciardi R et al., 2020 | 30  (24/80) | 92  (24/26) |  |  | 29  (5/17) | 19  (5/26) | 27  (17/63) | 65  (17/26) | 40  (12/30) | 46  (12/26) | 56  (9/16) | 35  (9/26) | 44  (4/9) | 15  (4/26) | 67  (10/15) | 38  (10/26) |  |  |
| Javanian M et al., 2020 | 24  (12/51) | 63  (12/19) |  |  |  |  | 38  (12/32) | 63  (12/19) | 27  (10/37) | 53  (10/19) | 40  (8/20) | 42  (8/19) | 42  (5/12) | 26  (5/19) | 42  (5/12) | 26  (5/19) | 67  (2/3) | 11  (2/19) |
| Nowak B et al., 2020 | 34  (30/87) | 65  (30/46) |  |  |  |  | 34  (27/80) | 59  (27/46) | 50  (16/32) | 35  (16/46) | 38  (22/58) | 48  (22/46) | 46  (6/13) | 13  (6/46) | 23  (8/35) | 17  (8/46) |  |  |
| Richardson S et al., 2020 | 22  (337/1499) | 61  (337/553) | 38  (419/1095) | 76  (419/553) |  |  | 13  (384/3026) | 69  (384/553) | 12  (224/1808) | 41  (224/553) |  |  |  |  |  |  |  |  |
| Tomlins J et al., 2020 | 20  (12/60) | 60  12/20) |  |  |  |  | 31  (11/35) | 55  (11/20) | 35  (13/37) | 65  (13/20) | 37  (7/19) | 35  (7/20) | 40  (4/10) | 20  (4/20) | 27  (6/22) | 30  (6/20) |  |  |
| Wu C et al., 2020 | 23  (29/128) | 66  (29/44) |  |  |  |  | 41  (16/39) | 36 (16/44) | 50  (11/22) | 25 (11/44) | 50  (4/8) | 9  (4/44) |  |  |  |  |  |  |
| Yao Q et al., 2020 | 16  (7/43) | 58  (7/12) | 35  (6/17) | 50  (6/12) | 75  (3/4) | 25  (3/12) | 44  (7/16) | 58  (7/12) | 20  (1/5) | 8  (1/12) | 50  (2/4) | 17  (2/12) |  |  |  |  | 50  (1/2) | 8  (1/12) |
| Zhang G et al., 2020 | 30  (16/53) | 64  (16/25) | 41  (7/17) | 28  (7/25) |  |  |  |  |  |  |  |  |  |  |  |  |  |  |
| Zhou F et al., 2020 | 32  (38/119) | 70  (38/54) |  |  | 45 (5/11) | 9 (5/54) | 45 (26/58) | 48 (26/54) | 47  (17/36) | 31 (17/54) | 87 (13/15) | 24 (13/54) | 67 (4/6) | 7 (4/54) | 0 (0/2) | 0 (0/54) |  |  |
| Turcotte J et al., 2020 | 42 (26/62) | 54 (26/48) |  |  | 42 (18/43) | 38 (18/48) | 45 (35/77) | 73 (35/48) | 61 (28/46) | 58 (28/48) | 54 (14/26) | 29 (14/48) | 41 (14/34) | 29 (14/48) | 74 (20/27) | 42 (20/48) |  |  |
| Zhang J et al., 2020 | 5 (15/321) | 60 (15/25) | 6 (19/315) | 76 (19/25) |  |  |  |  |  |  | 10 (16/164) | 64 (16/25) | 15 (9/60) |  |  |  |  |  |
| Nikpouraghdam M. 2020 | 9 (167/1955) | 70 (167/239) | 14 (160/1164) | 67 (160/239) |  |  | 14 (8/59) | 3 (8/239) | 10 (11/113) | 5 (11/239) | 11 (4/37) | 2 (4/239) | 30 (6/20) | 4 (9/239) | 17 (3/18) | 1 (3/239) |  |  |
| Khalil K 2020 | 32 (42/130) | 72 (42/58) | 42 (49/117) | 84 (49/58) | 32 (28/88) | 48 (28/58) | 37 (37/99) | 64 (37/58) | 34 (21/61) | 36 (21/58) | 46 (11/24) | 19 (11/58) | 23 (28/122) | 10 (6/58) | 75 (12/16) | 21 (12/58) | 83 (5/6) | 9 (5/58) |
| Iaccarino G 2020 | 12 (125/1018) | 67 (125/188) |  |  |  |  | 16 (137/873) | 73 (137/188) | 23 (60/269) | 32 (60/188) | 26 (56/216) | 30 (56/188) | 11 (16/146) | 15 (28/188) | 36 (31/87) | 17 (31/188) |  |  |
| Hsu H 2020 | 5 (66/1312) | 67 (66/98) | 9 (80/928) | 82 (80/98) |  |  | 6 (71/1248) | 72 (71/98) | 6 (46/708) | 47 (46/98) | 16 (30/190) | 31 (30/98) | 15 (9/60) | 16 (16/98) | 10 (44/438) | 45 (44/98) |  |  |
| Giacomelli A 2020 | 13 (9/72) | 19 (9/48) | 34 (34/99) | 71 (34/48) | 24 (17/70) | 35 (17/48) |  |  |  | 34 (45/131) |  |  |  |  |  |  |  |  |
| Garibaldi B 2020 | 17 (76/443) | 58 (76/131) | 0 (NR/) | 0 (NR/) | 19 (46/239) | 35 (46/131) | 22 (87/389) | 66 (87/131) | 18 (45/252) |  | 27 (71/262) |  | 17 (26/151) | 20 (26/131) | 34 (36/106) | 27 (36/131) | 9 (3/34) | 2 (3/131) |
| Hewitt J et al., 2020 | 28 (255/903) | 60 (255/425) | 34 (370/  1076) | 87 (370/425) | 29 (211/724) | 50 (211/425) | 30 (238/804) | 56 (238/425) | 27 (415/1564) | 30 (128/425) | 38 (132/345) | 31 (132/425) |  |  |  |  |  |  |
| Escalera-Antezana J et al., 2020 | 4 (2/57) | 33 (2/6) | 16 (5/31) | 83 (5/6) |  |  | 23 (3/13) | 50 (3/6) | 7 (7/107) | 33 (2/6) | 33 (1/3) | 17 (1/6) |  |  |  |  |  |  |
| Chilimuri S et al., 2020 | 47 (112/236) | 70 (112/160) |  |  |  |  | 51 (115/225) | 72 (115/160) | 47 (175/375) | 56 (90/160) | 61 (38/62) | 24 (38/160) | 47 (29/62) | 18 (29/160) | 61 (31/51) | 19 (31/160) | 61 (11/18) | 7 (11/160) |
| Pellaud et al., 2020 | 20 (24/119) | 73 (24/33) |  |  | 20 (11/56) | 33 (11/33) | 19 (22/118) | 67 (22/33) | 27 (52/196) | 30 (10/33) | 38 (10/26) | 30 (10/33) | 25 (4/16) | 12 (4/33) |  |  |  |  |
| Brill S et al., 2020 | 41 (111/272) | 64 (111/173) | 51 (161/313) | 93 (161/173) |  |  | 46 (90/195) | 52 (90/173) | 30 (134/450) | 31 (53/173) | 55 (78/141) | 45 (78/173) |  |  |  |  |  |  |
| Rivera-Izquierdo et al., 2020 | 29 (38/131) | 62 (38/61) | 40 (58/144) | 95 (58/61) | 26 (16/62) | 26 (16/61) | 41 (47/116) | 77 (47/61) | 22 (52/238) | 44 (27/61) | 67 (36/54) | 59 (36/61) |  |  | 65 (15/23) | 25 (15/61) |  |  |
| Shahriarirad R et al., 2020 | 7 (5/71) | 56 (5/9) | 11 (3/28) | 33 (3/9) |  |  | 9 (2/22) | 22 (2/9) | 14 (16/113) | 22 (2/9) | 13 (2/16) | 22 (2/9) | 11 (1/9) | 11 (1/9) | 0 (0/6) | 0 (0/9) |  |  |
| Gregoriano et al., 2020 |  |  |  |  |  |  |  |  | 22 (22/99) |  |  |  |  |  |  |  |  |  |
| Khamis F et al., 2020 |  |  |  |  |  |  |  |  | 32 (20/63) |  |  |  |  |  |  |  |  |  |
| Liu S et al., 2020 |  |  |  |  |  |  |  |  | 6 (40/  625) |  |  |  |  |  |  |  |  |  |
| Tambe et al., 2020 |  |  |  |  |  |  | 53 (32/60) | 55 (32/58) | 67 (28/42) | 48 (28/58) | 50 (2/4) | 3 (2/58) | 60 (6/10) | 10 (6/58) | 0 (2/2) | 3 (2/58) | 0 (4/4) | 7 (4/58) |
| Okoh et al., 2020 | 47 (60/129) | 62 (60/97) |  |  |  |  | 44 (77/175) | 79 (77/97) | 43 (50/115) | 52 (50/97) | 59 (29/49) | 30 (29/97) | 43 (10/23) | 10 (10/97) | 63 (29/46) | 30 (29/97) |  |  |
| Lendorf et al., 2020 | 16 (11/67) | 69 (11/16) |  |  |  |  |  |  |  |  |  |  |  |  |  |  |  |  |
| Ciceri et al., 2020 | 23 (70/299) | 74 (70/95) | 37 (77/207) | 81 (77/95) |  |  | 32 (65/203) | 68 (65/95) | 32 (22/69) | 23 (22/95) | 49 (25/51) | 26 (25/95) | 73 (16/22) | 17 (16/95) | 51 (24/47) | 25 (24/95) |  |  |

Note: cells are shaded for no data in the study.
