# Supplementary material for: Prevalence and predictors of death and severe disease in patients hospitalized due to COVID-19: A comprehensive systematic review and meta-analysis of 77 studies and 38,000 patients
Source: PLoS One. 2020 Dec 7;15(12):e0243191. doi: 10.1371/journal.pone.0243191 (PMC7721151; doi:10.1371/journal.pone.0243191)
Supplement: S3 Table — (DOCX) [file pone.0243191.s003.docx]

S3 Table. Prevalence of severe disease in risk groups and prevalence of risk factor in patients with severe COVID-19 during hospitalization (Dec 2019-May 2020)

| Author, year | Male sex, % (n/N) | | Age≥60  % (n/N) | | Smoking history (SH),  % (n/N) | | Hypertension (HTN), % (n/N) | | Diabetes mellitus (DM),  % (n/N) | | Cardiovascular disease (CVD),  % (n/N) | | Chronic Obstructive Pulmonary Disease (COPD),  % (n/N) | | Chronic Kidney Disease (CKD),  % (n/N) | | Chronic liver disease,  % (n/N) | |
| --- | --- | --- | --- | --- | --- | --- | --- | --- | --- | --- | --- | --- | --- | --- | --- | --- | --- | --- |
|  | Severe disease in male/Total male | Severe disease in male/Total Severe disease | Severe disease in >60/  Total>60 | Severe disease in >60/  Total Severe disease | Severe disease in SH/  Total SH | Severe disease in SH/Total Severe disease | Severe disease in HTN/Total HTN | Severe disease in HTN/Total Severe disease | Severe disease in DM/Total DM | Severe disease in DM/Total Severe disease | Severe disease in CVD/Total CVD | Severe disease in CVD/Total CVD | Severe disease in COPD/Total COPD | Severe disease in COPD /Total COPD | Severe disease in CKD/Total CKD | Severe disease in CKD/Total CKD | Severe disease in CLD/Total CLD | Severe disease in CLD/Total CLD |
| Aggarwal S et al., 2020 | 42  (5/12) | 63  (5/8) |  |  | 0  (0/0) | 0  (0/8) | 33  (3/9) | 38  (3/8) | 40  (2/5) | 25  (2/8) | 71  (5/7) | 63  (5/8) | 50  (1/2) | 13  (1/8) | 50  (3/6) | 38  (3/8) |  |  |
| Cao J et al.,  2020 | 0  (0/126) |  |  |  |  |  |  |  |  |  |  |  |  |  |  |  |  |  |
| CDC (USA), 2020 |  |  | 32  (232/715) | 55  (232/424) | 36  (38/105) | 8  (38/457) |  |  | 67  (148/399) | 32  (148/457) | 35  (132/374) | 29  (132/457) | 38  (94/246) | 21  (94/457) | 37  (56/151) | 12  (56/457) | 44  (7/16) | 2  (7/457) |
| Chen G et al., 2020 | 59  (10/17) | 91  (10/11) | 67  (10/15) | 91  (10/11) |  |  | 80  (4/5) | 36  (4/11) | 67  (2/3) | 18  (2/11) |  |  |  |  |  |  |  |  |
| Chen Q et al., 2020 | 29  (23/79) | 53  (23/43) |  |  | 20  (3/15) | 7  (3/43) | 43  (9/21) | 21  (9/43) | 50  (7/14) | 16  (7/43) | 100  (1/1) | 2  (1/43) | 0  (0/6) | 0  (0/43) | 33  (1/3) | 2  (1/43) | 67  (4/6) | 9  (4/43) |
| Chen T et al., 2020 | 84  (49/58) | 66  (49/74) |  |  |  |  |  |  |  |  |  |  |  |  |  |  |  |  |
| Feng Y et al., 2020 | 30  (81/271) | 65  (81/124) | 38  (45/118) | 36  (45/124) | 39  (17/44) | 14  (17/124) | 64  (72/113) | 58  (72/124) | 35  (17/49) | 14  (17/124) | 45  (17/38) | 14  (17/124) | 64  (14/22) | 11  (14/124) | 50  (2/4) | 2  (2/124) |  |  |
| Ferguson J et al., 2020 | 34  (13/38) | 62  (13/21) |  |  | 35  (6/17) | 29  (6/21) | 42  (11/26) | 52  (11/21) | 50  (10/20) | 48  (10/21) | 29  (2/7) | 10  (2/21) | 30  (3/10) | 14  (3/21) | 22  (2/9) | 10  (2/21) |  |  |
| Gold J et al., 2020 |  |  | 41  (48/117) | 56  (48/86) |  |  |  |  |  |  |  |  |  |  |  |  |  |  |
| Goyal S et al., 2020 | 39  (92/238) | 71  (92/130) |  |  | 30  (6/20) | 5  (6/130) | 36  (70/197) | 54  (70/130) | 36  (36/99) | 28  (36/130) | 46  (25/54) | 19  (25/130) | 35  (7/20) | 5  (7/130) |  |  |  |  |
| Guan W et al., 2020 | 16  (100/637) | 58  (100/173) | 29  (44/153) | 27  (44/163) | 24  (38/158) | 22  (38/172) | 25  (41/165) | 24  (41/173) | 35  (28/81) | 16  (28/173) | 37  (10/27) | 6  (10/173) | 50  (6/12) | 3  (6/173) | 38  (3/8) | 4  (3/67) | 4  (1/23) | 1  (1/173) |
| Guan Wei-Jie, 2020 (ERJ) |  |  |  |  |  |  | 20  (53/269) | 40  (53/131) | 10  (13/130) | 10  (13/131) | 22  (13/59) | 10  (13/131) | 50  (12/24) | 9  (12/131) | 29  (6/21) | 5  (6/131) | 11  (3/28) | 2  (3/131) |
| Hu L et al., 2020 | 55  (91/166) | 53  (91/172) | 70  (78/111) | 45  (78/172) | 68  (26/38) | 15  (26/172) | 63  (66/105) | 38  (66/172) | 70  (33/47) | 19  (33/172) | 80  (33/41) | 19  (33/172) | 100  (6/6) | 3  (6/172) | 43  (3/7) | 2  (3/172) | 40  (2/5) | 1  (2/172) |
| Huang C et al., 2020 | 37  (11/30) | 85  (11/13) |  |  | 0  (0/3) | 0  (0/13) | 33  (2/6) | 15  (2/13) | 13  (1/8) | 8  (1/13) | 50  (3/6) | 23  (3/13) |  |  |  |  |  |  |
| Li X et al., 2020 | 55  (153/279) | 57  (153/169) | 64  (135/210) | 50  (135/269) | 55  (51/92) | 19  (51/265) | 63  (104/166) | 39  (104/269) | 63  (52/83) | 19  (52/269) | 82  (28/34) | 10  (28/269) | 76  (13/17) | 5  (13/269) | 60  (6/10) | 2  (6/269) |  |  |
| Liu W et al., 2020 | 18  (7/39) | 64  (7/11) |  |  | 60  (3/5) | 27  (3/11) | 25  (2/8) | 18  (2/11) | 40  (2/5) | 18  (2/11) | 50  (1/2) | 9  (1/11) |  |  |  |  |  |  |
| Richardson S et al., 2020 |  |  | 18  (200/1095) | 54  (200/373) |  |  |  |  |  |  |  |  |  |  |  |  |  |  |
| Shi Y et al., 2020 | 14  (36/259) | 73  (36/49) |  |  | 15  (6/40) | 12  (6/49) | 26  (26/99) | 53  (26/49) | 24  (7/29) | 18  (7/40) | 36  (4/11) | 10  (4/40) |  |  | 29  (2/7) | 5  (2/40) | 9  (2/22) | 5  (2/40) |
| Sun L et al., 2020 | 26  (8/31) | 53  (8/15) | 80  (8/10) | 53  (8/15) |  |  | 75  (6/8) | 40  (6/15) | 40  (2/5) | 13  (2/15) | 100  (1/1) | 7  (1/15) |  |  | 100  (1/1) | 7  (1/15) | 67  (2/3) | 13  (2/15) |
| Tian S et al., 2020 | 20  (26/127) | 57  (26/46) | 42  (20/48) | 43  (20/46) |  |  |  |  |  |  |  |  |  |  |  |  |  |  |
| Wan S et al., 2020 | 29  (21/72) | 53  (21/40) |  |  | 11  (1/9) | 3  (1/40) | 31  (4/13) | 10  (4/40) | 75  (9/12) | 23  (9/40) | 86  (6/7) | 15  (6/40) |  |  |  |  | 50  (1/2) | 3  (1/40) |
| Wang D et al., 2020 | 29  (22/75) | 61  (22/36) |  |  |  |  | 49  (21/43) | 58  (21/36) | 57  (8/14) | 22  (8/36) | 45  (9/20) | 25  (9/36) | 75  (3/4) | 8  (3/36) | 50  (2/4) | 6  (2/36) | 0  (0/4) | 0  (0/36) |
| Wang R et al., 2020 | 23  (16/71) | 64  (16/25) |  |  | 44  (7/16) | 28  (7/25) |  |  |  |  |  |  |  |  |  |  |  |  |
| Wang Z et al., 2020 | 22  (7/32) | 50  (7/14) |  |  |  |  | 56  (5/9) | 36  (5/14) | 86  (6/7) | 43  (6/14) | 63  (5/8) | 36  (5/14) | 50  (2/4) | 14  (2/14) |  |  | 0  (0/1) | 0  (0/14) |
| Wu C et al., 2020 | 47  (60/128) | 71  (60/84) |  |  |  |  | 59  (23/39) | 27  (23/84) | 73  (16/22) | 19  (16/84) | 63  (5/8) | 6  (5/84) |  |  |  |  |  |  |
| Yao Q et al., 2020 | 14  (6/43) | 46  (6/13) | 18  (3/17) | 23  (3/13) | 0  (0/4) | 0  (0/12) | 13  (2/16) | 15  (2/13) | 40  (2/5) | 15  (2/13) | 0  (0/4) | 0  (0/13) |  |  |  |  | 0  (0/2) | 0  (0/13) |
| Young BE et al., 2020 | 22  (2/9) | 33  (2/6) |  |  |  |  |  |  |  |  |  |  |  |  |  |  |  |  |
| Yu X et al., 2020 | 12  (20/172) | 77  (20/26) | 20  (21/107) | 81  (21/26) | 8  (2/26) | 8  (2/26) | 14  (9/64) | 35  (9/26) | 18  (5/28) | 19  (5/26) | 33  (8/24) | 31  (8/26) |  |  |  |  |  |  |
| Zhang G et al., 2020 | 40  (21/53) | 66  (21/32) | 41  (7/17) | 22  (7/32) |  |  |  |  |  |  |  |  |  |  |  |  |  |  |
| Zhang JJ et at., 2020 | 46  (33/71) | 57  (33/58) | 34  (20/58) | 34  (20/58) | 67  (6/9) | 10  (6/58) | 52  (22/42) | 38  (22/58) | 47  (8/17) | 14  (8/58) | 57  (4/7) | 7  (4/58) | 100  (2/2) | 3  (2/58) | 100  (2/2) | 3  (2/58) | 50  (4/8) | 7  (4/58) |
| Zhao X-Y et al., 2020 | 29  (14/49) | 47  (14/30) | 50  (8/16) | 27  (8/30) |  |  |  |  | 33  (1/3) | 3  (1/30) |  |  |  |  | 100  (1/1) | 3  (1/30) |  |  |
| Zheng S et al., 2020 |  |  |  |  |  |  | 89  (31/35) | 42  (31/74) | 91  (10/11) | 14  (10/74) | 100  (7/7) | 9  (7/74) | 100  (4/4) | 5  (4/74) | 100  (1/1) | 1  (1/74) | 67  (2/3) | 3  (2/74) |
| Zheng Y et al., 2020 |  |  |  |  | 25  (2/8) | 7  (2/30) |  |  | 25  (1/4) | 3  (1/30) |  |  |  |  |  |  |  |  |
| Zhang J et al., 2020 | 64 (205/321) | 50 (205/409) | 78 (246/315) | 60 (246/409) |  |  |  |  |  |  | 80 (131/164) | 32 (131/409) |  |  |  |  |  |  |
| Kalligeros M 2020 | 46 (29/63) | 66 (29/44) |  |  | 42 (20/48) | 45 (20/44) | 47 (31/66) | 70 (31/44) | 55 (21/38) | 48 (21/44) | 56 (14/25) | 32 (14/44) | 55 (11/20) | 25 (11/44) | 36 (4/11) | 9 (4/44) | 0 (0/44) | 0 (0/44) |
| Cao Z 2020 | 42 (16/38) | 59 (16/27) |  |  | 80 (4/5) | 15 (4/27) | 20 (4/20) | 15 (4/27) | 50 (3/6) | 11 (3/27) | 50 (5/10) | 19 (5/27) | 0 (0/5) | 0 (0/27) |  |  |  |  |
| Hsu H 2020 | 10 (127/1312) | 68 (127/188) | 10 (91/928) | 48 (91/188) |  |  | 8 (105/1248) | 56 (105/188) | 10 (71/708) | 38 (71/188) | 8 (16/190) | 9 (16/188) | 12 (17/146) | 9 (17/188) | 11 (46/438) | 24 (46/188) |  |  |
| Hur K 2020 | 32 (88/271) | 64 (88/138) | 38 (83/217) | 60 (83/138) | 34 (55/163) | 40 (55/138) | 31 (82/267) | 59 (82/138) | 35 (56/160) | 41 (56/138) | 36 (40/111) | 29 (40/138) | 29 (23/78) | 17 (23/138) | 29 (12/42) | 9 (12/138) |  |  |
| Shabrawishi M 2020 | 14 (13/90) | 81 (13/16) |  |  |  |  | 7 (3/42) | 19 (3/16) | 18 (7/38) | 44 (7/16) | 27 (3/11) |  | 0 (1/1) | 6 (1/16) | 40 (4/10) | 25 (4/16) | 0 (1/16) | 6 (1/16) |
| Garibaldi B 2020 | 22 (96/443) | 56 (96/171) | 0 (NR/) | 0 (NR/) | 24 (58/239) | 34 (58/171) | 18 (71/389) | 42 (71/171) | 22 (56/252) | 33 (56/171) | 24 (64/266) |  | 19 (28/151) |  | 22 (23/106) | 13 (23/171) | 44 (15/171) | 9 (15/171) |
| Zhan et al., 2020 | 39 (73/186) | 49 (73/148) | 48 (105/221) | 71 (105/148) | 54 (25/46) | 17 (25/148) |  |  | 52 (46/88) | 31 (46/148) | 49 (77/156) |  | 77 (24/31) |  | 57 (16/28) | 11 (16/148) |  |  |
| Wei et al., 2020 | 6 (10/155) | 71 (10/14) | 18 (11/60) | 79 (11/14) | 7 (2/28) | 14 (2/14) | 17 (8/47) | 57 (8/14) | 14 (2/14) | 14 (2/14) | 33 (4/12) |  | 29 (2/7) |  |  |  |  |  |
| Sun L et al., 2020 | 26 (8/31) | 53 (8/15) |  |  |  |  |  |  |  |  |  |  |  |  |  |  |  |  |
| Khamis F et al., 2020 | 40 (21/53) | 88 (21/24) |  |  | 25 (1/4) | 4 (1/24) | 35 (7/20) | 29 (7/24) | 55 (11/20) | 46 (11/24) | 50 (2/4) | 8 (2/24) |  |  | 25 (1/4) | 4 (1/24) |  |  |
| Gregoriano et al., 2020 | 45 (28/62) | 80 (28/35) |  |  | 33 (2/6) | 8 (2/24) | 34 (19/56) | 54 (19/35) | 36 (8/22) | 23 (8/35) | 32 (9/28) | 26 (9/35) | 57 (4/7) | 11 (4/35) | 36 (10/28) | 29 (10/35) |  |  |
| Pellaud et al., 2020 | 29 (34/119) | 69 (34/49) | 23 (34/148) | 69 (34/49) | 32 (18/56) | 37 (18/49) | 23 (27/118) | 55 (27/49) | 21 (11/52) | 22 (11/49) | 23 (6/26) | 12 (6/49) | 31 (5/16) | 10 (5/49) |  |  |  |  |
| Liu S et al., 2020 | 12 (41/329) | 64 (41/64) | 33 (26/79) | 41 (26/64) |  |  | 21 (19/91) | 30 (19/64) | 25 (10/40) | 16 (10/64) |  |  |  |  |  |  |  |  |
| Shahriarirad R et al., 2020 | 10 (7/71) | 64 (7/11) | 11 (3/28) | 27 (3/11) |  |  | 23 (5/22) | 45 (5/11) | 13 (2/16) | 18 (2/11) | 25 (4/16) | 36 (4/11) | 11 (1/9) | 9 (1/11) | 0 (0/6) | 0 (0/11) |  |  |
| Suleyman et al., 2020 | 48 (80/165) | 57 (80/141) | 46 (92/202) | 65 (92/141) | 42 (58/137) | 41 (58/141) | 43 (111/258) | 79 (111/141) | 47 (73/156) | 52 (73/141) | 46 (26/56) | 18 (26/141) | 44 (18/41) | 13 (18/141) | 52 (83/161) | 59 (83/141) |  |  |
| Shekhar et al., 2020 | 65 (15/23) | 44 (15/34) | 64 (9/14) | 26 (9/34) |  |  | 82 (14/17) | 41 (14/34) | 89 (16/18) | 47 (16/34) |  |  |  |  |  |  | 0 (2/34) | 6 (2/34) |
| Lendorf et al., 2020 | 25 (17/67) | 85 (17/20) |  |  | 16 (7/43) | 35 (7/20) | 24 (9/38) | 45 (9/20) | 44 (7/16) | 35 (7/20) | 16 (3/19) | 15 (3/20) | 0 (0/8) | 0 (0/20) | 38 (3/8) | 15 (3/20) |  |  |
| Jang et al., 2020 | 29 (14/48) | 61 (14/23) |  |  |  |  | 32 (12/37) | 52 (12/23) | 48 (14/29) | 61 (14/23) | 10 (1/10) | 4 (1/23) | 50 (2/4) | 9 (2/23) |  |  | 0 (0/23) | 0 (0/23) |
| Argenziano et al., 2020 | 31 (158/511) | 67 (158/236) | 26 (101/388) | 43 (101/236) | 30 (59/198) | 25 (59/236) | 30 (158/525) | 67 (158/236) | 30 (101/333) | 43 (101/236) | 25 (29/115) | 12 (29/236) | 25 (14/56) | 6 (14/236) | 22 (27/125) | 11 (27/236) | 29 (5/236) | 2 (5/236) |

Note: cells are shaded for no data in the study.
