# Supplementary material for: Prevalence and predictors of death and severe disease in patients hospitalized due to COVID-19: A comprehensive systematic review and meta-analysis of 77 studies and 38,000 patients
Source: PLoS One. 2020 Dec 7;15(12):e0243191. doi: 10.1371/journal.pone.0243191 (PMC7721151; doi:10.1371/journal.pone.0243191)
Supplement: S4 Table — #Award of Points: Selection: points were awarded based on representativeness of the exposed group and unexposed group (2 points), ascertainment of exposures (1 point), and demonstration that outcome of interest was not present at the start of the study (1 point). Comparability (2 points): points were awarded based on whether the analyses were adjusted for age, sex, and other risk factors (2 points for adjustment to age and sex). Outcome (3points): points were awarded based on ascertainment of outcome through record linkage or independent blind assessment (1 points); duration of follow-up (1 point) (hospitalization till discharge); and adequacy of follow up for study population (complete follow up for the patients (vs whether patients were currently under treatment at the time of study report) (1 point), or if the patients currently under admission are excluded from outcome assessment (1 point). (DOCX) [file pone.0243191.s004.docx]

**S4 Table**

**Newcastle-Ottawa quality assessment (modified) for studies^#^**

|  | **Cohort studies** |  |  |  |  |  |
| --- | --- | --- | --- | --- | --- | --- |
| S. No. | Author, Year | Study Design | Selection  (out of 4) | Comparability (out of 2) | Outcome  (out of 3) | Total |
| 1 | Aggarwal S et al., 2020 | Retrospective | 2 | 1 | 2 | 5 |
| 2 | Argenziano et al., 2020 | Retrospective | 4 | 1 | 1 | 6 |
| 3 | Brill S et al., 2020 | Retrospective | 3 | 0 | 2 | 5 |
| 4 | Cao Z et al., 2020 | Retrospective | 2 | 0 | 2 | 4 |
| 5 | CDC, USA | Retrospective | 3 | 1 | 2 | 6 |
| 6 | Chen G et al., 2020 | Retrospective | 2 | 1 | 2 | 5 |
| 7 | Chen J et al., 2020 | Retrospective | 3 | 2 | 2 | 7 |
| 8 | Chen Q et al., 2020 | Retrospective | 2 | 0 | 3 | 5 |
| 9 | Chen T et al., 2020 | Retrospective | 3 | 0 | 2 | 5 |
| 10 | Chilimuri S et al., 2020 | Retrospective | 4 | 2 | 2 | 8 |
| 11 | Ciceri et al., 2020 | Retrospective | 4 | 2 | 2 | 8 |
| 12 | Cummings MJ et al., 2020 | Prospective | 3 | 2 | 2 | 7 |
| 13 | Deng Y et al., 2020 | Retrospective | 3 | 1 | 2 | 6 |
| 14 | Du R-H et al., 2020 | Retrospective | 2 | 2 | 2 | 6 |
| 15 | Feng Y et al., 2020 | Retrospective | 3 | 2 | 2 | 7 |
| 16 | Ferguson J et al., 2020 | Retrospective | 2 | 1 | 2 | 5 |
| 17 | Galloway et al., 2020 | Retrospective | 4 | 2 | 2 | 8 |
| 18 | Garibaldi B et al., 2020 | Retrospective | 3 | 2 | 2 | 7 |
| 19 | Giacomelli, A et al., 2020 | Retrospective | 3 | 2 | 2 | 7 |
| 20 | Gregoriano C et al., 2020 | Retrospective | 3 | 1 | 2 | 6 |
| 21 | Goyal P et al. 2020 | Retrospective | 3 | 0 | 2 | 5 |
| 22 | Gold J et al, 2020 | Retrospective | 3 | 2 | 2 | 7 |
| 23 | Guan et al., 2020 | Retrospective | 3 | 0 | 2 | 5 |
| 24 | Guan Wei-Jie, 2020 | Retrospective | 3 | 2 | 2 | 7 |
| 25 | Hsu H et al., 2020 | Retrospective | 3 | 0 | 2 | 5 |
| 26 | Hu L et al., 2020 | Retrospective | 3 | 2 | 2 | 7 |
| 27 | Hur K et al., 2020 | Retrospective | 3 | 2 | 3 | 8 |
| 28 | Huang C et al., 2020 | Prospective | 4 | 1 | 3 | 8 |
| 29 | Hewitt J et al., 2020 | Retrospective | 4 | 2 | 3 | 9 |
| 30 | Inciardi R et el., 2020 | Retrospective | 2 | 1 | 3 | 6 |
| 31 | Jang et al., 2020 | Retrospective | 3 | 1 | 1 | 5 |
| 32 | Javanian M et al., 2020 | Retrospective | 2 | 2 | 3 | 7 |
| 33 | Kalligeros, M et al., 2020 | Retrospective | 2 | 2 | 3 | 7 |
| 34 | Khamis F et al., 2020 | Retrospective | 2 | 0 | 1 | 3 |
| 35 | Khalil, K et al., 2020 | Retrospective | 2 | 2 | 2 | 6 |
| 36 | Lendorf et al., 2020 | Retrospective | 3 | 1 | 2 | 6 |
| 37 | Liu W et al., 2020 | Retrospective | 2 | 2 | 3 | 7 |
| 38 | Liu S et al., 2020 | Retrospective | 3 | 2 | 3 | 8 |
| 39 | Li X et al., 2020 | Retrospective | 3 | 2 | 2 | 7 |
| 40 | Nikpouraghdam, M et al., 2020 | Retrospective | 3 | 2 | 2 | 7 |
| 41 | Nowak B et al., 2020 | Retrospective | 2 | 0 | 2 | 4 |
| 42 | Okoh et al., 2020 | Retrospective | 2 | 2 | 2 | 6 |
| 43 | Pellaud C et al., 2020 | Retrospective | 3 | 0 | 2 | 5 |
| 44 | Palaiodimos L et al., 2020 | Retrospective | 3 | 2 | 2 | 7 |
| 45 | Richardson S et al., 2020 | Retrospective | 3 | 1 | 3 | 7 |
| 46 | Rivera-Izquierdo M et al., 2020 | Retrospective | 2 | 2 | 1 | 5 |
| 47 | Shabrawishi, M. et al., 2020 | Retrospecitve | 2 | 0 | 2 | 4 |
| 48 | Shariarirad R et al., 2020 | Retrospective | 3 | 0 | 1 | 4 |
| 49 | Shekhar et al., 2020 | Retrospective | 2 | 0 | 2 | 4 |
| 50 | Shi Y et al., 2020 | Retrospective | 1 | 2 | 2 | 5 |
| 51 | Suleyman et al., 2020 | Retrospective | 3 | 1 | 2 | 6 |
| 52 | Sun L et al., 2020 | Retrospective | 3 | 1 | 2 | 6 |
| 53 | Tian S et al., 2020 | Retrospective | 2 | 1 | 2 | 5 |
| 54 | Tomlins J et al., 2020 | Retrospective | 2 | 1 | 2 | 5 |
| 55 | Wan S et al., 2020 | Retrospective | 2 | 1 | 2 | 5 |
| 56 | Wang D et al., 2020 | Retrospective | 3 | 1 | 3 | 7 |
| 57 | Wang R et al., 2020 | Retrospective | 2 | 1 | 2 | 5 |
| 58 | Wang Z et al., 2020 | Retrospective | 2 | 1 | 2 | 5 |
| 59 | Wei et al., 2020 | Retrospective | 4 | 1 | 2 | 7 |
| 60 | Wu C et al., 2020 | Retrospective | 4 | 1 | 3 | 8 |
| 61 | Yao Q et al., 2020 | Retrospective | 3 | 1 | 2 | 6 |
| 62 | Yang X et al., 2020 | Retrospective | 2 | 0 | 2 | 4 |
| 63 | Young BE et al., 2020 | Retrospective | 2 | 1 | 2 | 5 |
| 64 | Yu X et al., 2020 | Retrospective | 2 | 2 | 2 | 6 |
| 65 | Zhang G et al., 2020 | Retrospective | 2 | 1 | 2 | 5 |
| 66 | Zhang JJ et al., 2020 | Retrospective | 2 | 1 | 2 | 5 |
| 67 | Zhao X-Y et al., 2020 | Retrospective | 2 | 1 | 2 | 5 |
| 68 | Zhan T et al., 2020 | Retrospective | 3 | 0 | 2 | 5 |
| 69 | Zheng S et al., 2020 | Retrospective | 2 | 1 | 2 | 5 |
| 70 | Zheng Y et al., 2020 | Retrospective | 2 | 1 | 3 | 6 |
| 71 | Zhou F et al., 2020 | Retrospective | 3 | 2 | 2 | 7 |
| 72 | Zhang J et al.,  2020 | Retrospective | 4 | 2 | 2 | 8 |
|  | **Cross-sectional studies** |  | Selection  (out of 4) | Comparability (out of 2) | Outcome  (out of 3) | Total  (out of 10) |
| 73 | Escalera-Antezana J et al., 2020 | Cross-sectional | 3 | 2 | 1 | 6 |
| 74 | Iaccarino, G et al., 2020 | Cross-sectional | 3 | 2 | 2 | 7 |
| 75 | Tambe et al., 2020 | Cross-sectional | 2 | 0 | 2 | 4 |
| 76 | Turcotte J.J et al., 2020 | Retrospective | 3 | 2 | 2 | 7 |
| 77 | Yu T et al., 2020 | Cross-sectional | 2 | 2 | 2 | 6 |

**^#^Award of Points:**

**Selection:** points were awarded based on representativeness of the exposed group and unexposed group (2 points), ascertainment of exposures (1 point), and demonstration that outcome of interest was not present at the start of the study (1 point).

**Comparability (2 points)**: points were awarded based on whether the analyses were adjusted for age, sex, and other risk factors (2 points for adjustment to age and sex).

**Outcome (3points)**: points were awarded based on ascertainment of outcome through record linkage or independent blind assessment (1 points); duration of follow-up (1 point) (hospitalization till discharge); and adequacy of follow up for study population (complete follow up for the patients (vs whether patients were currently under treatment at the time of study report) (1 point), or if the patients currently under admission are excluded from outcome assessment (1 point).
