# Supplementary material for: Prevalence and predictors of death and severe disease in patients hospitalized due to COVID-19: A comprehensive systematic review and meta-analysis of 77 studies and 38,000 patients
Source: PLoS One. 2020 Dec 7;15(12):e0243191. doi: 10.1371/journal.pone.0243191 (PMC7721151; doi:10.1371/journal.pone.0243191)
Supplement: Attachment — Submitted filename: Response_To_Reviewers.docx [file pone.0243191.s007.docx]

October 8, 2020.

The Editorial Board,

PLOS ONE

Dear Editor,

Please find our response to the feedback from peer review in blue. We thank the editors and reviewers for their critique that helped to improve the paper.

Editors’ comments:

1. Please ensure that your manuscript meets PLOS ONE's style requirements, including those for file naming.

We have formatted the manuscript according to PLOS ONE's style requirements

2. As research on this topic is proceeding at a fast pace, we would recommend that the search is updated to allow an up-to-date view of the topic.

We have updated the literature search including reviewing ~13,000 additional citations, 410 full texts, and including 33 additional studies for meta-analysis.

3. We note that you have indicated that data from this study are available upon request. PLOS only allows data to be available upon request if there are legal or ethical restrictions on sharing data publicly.

We have submitted all data with this resubmission as Supporting Information files (S1 Table, S2 Table, and S3 Table).

4. Thank you for stating the following at the end of your manuscript: 'Funding: Dr. Dorjee is supported by grants from private philanthropists; the Johns Hopkins Alliance for Healthier World (Grant # 80045453); National Institute of Allergy and Infectious Diseases of the National Institute of Health (Grant # K01AI148583); the United Nations STOP TB PARTNERSHIP TB REACH (Grant # 134126); and the Pittsfield Anti-tuberculosis Association (PATA).' We note that you have provided funding information that is not currently declared in your Funding Statement. However, funding information should not appear in the Acknowledgments section or other areas of your manuscript. We will only publish funding information present in the Funding Statement section of the online submission form. Please remove any funding-related text from the manuscript and let us know how you would like to update your Funding Statement. Currently, your Funding Statement reads as follows: 'The author(s) received no specific funding for this work. 'Please include your amended statements within your cover letter; we will change the online submission form on your behalf.

I have removed the funding information from the manuscript text and including in the cover letter. Kindly amend the funding information statement based on the statement in the cover letter.

5. Please include a copy of Table 3 which you refer to in your text.

Table 3 included as Table 3.

**Reviewers' comments:**

Reviewer #1: Authors showed results from systematic literature review regarding SARS-C0-V2 and outcomes. They used PRISMa methodology; the article is presented in an intelligible fashion and is written in standard English. In general the manuscript has typical content as for review with accepted style. My points are listed below.

Conclusions and limitations are acceptable.

-some typhos exist needing correction: e.g. once angiotensin begins with capital letter other time with small letter.

Typos are fixed and angiotensin is now spelled with a small a

- citations are missing journel pages, for the instance ref.6, 7.

We have corrected the citations. They were previously in pre-proof format and hence page numbers were not available.

**Reviewer # 2:**

Reviewer #2: In this manuscript the Authors present the results of a fixed- effects meta-analysis designed to investigate the association between clinical/epidemiological risk factors and progression to death in patients hospitalized due to COVID-19. A total of 44 studies were included in the analysis, comprising 20594 hospitalized patients mainly from China and US and small cohorts from Italy, UK, Iran and Singapore. Despite the heterogeneity of the studies and the lack of adjusted measures for disease severity or death, the rationale behind the study is interesting and overall the paper is original and well written. The comparison between the prevalence of comorbidities/risk factors in hospitalized COVID-19 patients and in the general population is well summarized.

Thank you.

We feel that the Authors should blunt the link with cardiovascular diseases as the statement “…suggesting either a tendency for SARS-CoV-2 to more effectively establish infection in cardiovascular patients or to cause more severe disease among them prompting hospitalization” (page 20) does not seem quite convincing. This also suggests that the Discussion should better elaborate on the proportion of asymptomatic individuals and the interactions of comorbidities.

We have revised our description of the relationship between cardiovascular disease and SARS-CoV-2 infection risk to blunt the causal link. We have also discussed our findings in the context of asymptomatic infection. Below is the revision we have made in the discussion (lines: 381-90).

“To this end, we found that the prevalence of smoking history (23%), hypertension (55%), diabetes (33%) and heart disease (17%) in the hospitalized COVID-19 patients in the US were substantially higher than the corresponding prevalence of smoking (14%),^112^ hypertension (29%),^113^ diabetes (13%)^114^ and heart disease (9%)^115^ in the general US population that could indirectly suggest an association between these comorbidities and risk of SARS-CoV-2 infection or disease progression. However, we note that if the prevalence of these comorbidities in the asymptomatic individuals with COVID-19 in the general population is similar to that of their prevalence in the non-COVID-19 general population, then this difference–the higher prevalence of comorbidities in the hospitalized patients compared to the general population–could simply imply a higher risk of symptomatic infection or hospitalization for individuals having SARS-CoV-2 infection.”

Sincerely,

Kunchok Dorjee, MD, PhD

Assistant Professor of Medicine
