# Supplementary material for: Prevalence and predictors of death and severe disease in patients hospitalized due to COVID-19: A comprehensive systematic review and meta-analysis of 77 studies and 38,000 patients
Source: PLoS One. 2020 Dec 7;15(12):e0243191. doi: 10.1371/journal.pone.0243191 (PMC7721151; doi:10.1371/journal.pone.0243191)
Supplement: Attachment — Submitted filename: Response_To_Reviewers.docx [file pone.0243191.s008.docx]

Reviewer #1: The authors responded to all queries sufficiently. In recent version it is publishable in PLOS one.

Thank you

Reviewer #2: In this Manuscript the Authors present a Systematic Review and Meta-analysis of prevalence and predictors of death and severe disease in patients hospitalized due to COVID-19. They performed a fixed-effects meta-analysis using Shore’s adjusted confidence intervals to address heterogeneity. The paper is technically sound, well presented and adequately discussed.
Occasional formatting errors should be fixed.

We have fixed the formatting errors. The formatting issues we have addressed are shown in the manuscript version in track changes.
